# Supplementary material for: Dietary practices, physical activity and social determinants of non-communicable diseases in Nepal: A systemic analysis
Source: PLoS One. 2023 Feb 6;18(2):e0281355. doi: 10.1371/journal.pone.0281355 (PMC9901760; doi:10.1371/journal.pone.0281355)
Supplement: S1 Checklist — (RTF) [file pone.0281355.s001.rtf]

Consolidated criteria for reporting qualitative studies (COREQ): 32-item checklist

No.  Item 
	Guide questions/description	Location in manuscript (Section- Page #; Line #)	
Domain 1: Research team and reﬂexivity 			
Personal Characteristics 			
1. Interviewer/facilitator	SS	Methods- 3; 92,102	
2. Credentials	SS- MPH, MHCom.
All others- PhD 	NA	
3. Occupation	SS- PhD Student
All others- Lecturer/Professor	Title Page- a 	
4. Gender	SS, JF, DWL, AV- Male
RP, AM, DL- Female	N/A	
5. Experience and training	SS have received his training in qualitative research during his bachelor and masters studies. All other researchers are academicians experienced in qualitative research in health sciences.	NA	
Relationship with participants 			
6. Relationship established	No. 	N/A	
7. Participant knowledge of the interviewer 	Participants were made aware about the purpose of the study and the research team through “simple to understand” information sheet. Participants were provided the information sheet and after they thoroughly reviewed the sheet and/or verbally explained by the researcher, they were requested to provide written consent if they intended to participate in the study.	Method- 5; 142-145	
8. Interviewer characteristics	Interviewer's (SS) qualification and experience were shared in the information sheet; 
All interviews and focus groups were done by the first author
	NA


Method- 3; 92,102	
Domain 2: study design 			
Theoretical framework 			
9. Methodological orientation and Theory 	Systemic Intervention Methodology; Case study method and causal loop diagram; Framework approach for thematic analysis guided by the study framework	Method- 2-5; 42-145	
Participant selection 			
10. Sampling	Purposive	Method- 2-3; 51-103	
11. Method of approach	Face-to-face; Email; Telephone	NA	
12. Sample size	Key informant interviews: 63
Focus Group: 12 (5-10 participants per FG) 	Method- 3; 75-103	
13. Non-participation	There was no drop out or refusal after agreeing to participate in the study. Only one key policy level participant working in Non-government sector did not agree to participate citing organizational reasons despite assuring him/her of confidentiality. 	NA	
Setting			
14. Setting of data collection	Data were collected in Nepal at policy, district and community levels. 
All key informant interviews were done in workplace; Focus group were conducted in communities either at Community halls or home of a participant	Method- 2-3; 51-72

NA	
15. Presence of non-participants	No	NA	
16. Description of sample	Data were collected from different levels: policy, district and community level involving multi-sector participants as well as community level participants. Where essential, local authorities were involved to identify the potential participants. For FGs, we selected one disadvantaged and one advantaged/mixed communities in each of the selected VDCs/municipalities	Method- 2-4; 51-103

	
Data collection 			
17. Interview guide	The Interviews and FGs guidelines included semi-structured open ended questions reviewed by the research team. No piloting was done.	Method- 3; 75-103	
18. Repeat interviews	No	NA	
19. Audio/visual recording	The interviews and focus groups were audio recorded. Three interviewees did not consent to audio recording for personal reasons.	Method- 4; 106	
20. Field notes	No	NA	
21. Duration	The semi-structured interview ranged from 30-60 minutes; FGs ranged from 45-60 minutes	Method-3,4; 92, 103	
22. Data saturation	No. Data saturation was not considered as the study aimed to explore the range of stakeholder's perspective around the research question.	Method-3; 83-103	
23. Transcripts returned	No	N/A	
Domain 3: analysis and ﬁndings 			
Data analysis 			
24. Number of data coders	One: SS	NA	
25. Description of the coding tree	Coding and thematic analysis based on framework approach. The study framework guided the analysis.	Method- 4; 105-112
Fig 1
	
26. Derivation of themes	Themes were derived from the data	Method- 4; 105-112	
27. Software	Excel, Dedoose, Vensim	Method-4; 126-129	
28. Participant checking	Yes; Stakeholders validation was conducted at policy and district levels.	Method-4,5; 131-137	
Reporting 			
29. Quotations presented	Yes, themes were illustrated by presenting participants quotations. Due to confidentiality reasons, we have only identified the geographical location and level of the participants in the quotes.	Results- 5-10	
30. Data and ﬁndings consistent	Yes. This paper is part of the broader study on the social determinants of NCDs and primarily reports only on the dietary practice and physical activity and NCDs.	NA	
31. Clarity of major themes	Yes	Results- 5-12	
32. Clarity of minor themes	Minority themes has not been extensively discussed.      	NA	
