# Supplementary material for: Dietary practices, physical activity and social determinants of non-communicable diseases in Nepal: A systemic analysis
Source: PLoS One. 2023 Feb 6;18(2):e0281355. doi: 10.1371/journal.pone.0281355 (PMC9901760; doi:10.1371/journal.pone.0281355)
Supplement: Attachment — Submitted filename: PONE-D-21-14408_diet paper.pdf [file pone.0281355.s004.pdf]

PLOS ONE

Dietary practices, physical activity and social determinants of non-communicable diseases in Nepal: a systemic analysis

--Manuscript Draft--

**Manuscript Number:**	PONE-D-21-14408R1
**Article Type:**	Research Article
**Full Title:**	Dietary practices, physical activity and social determinants of non-communicable diseases in Nepal: a systemic analysis
**Short Title:**	Dietary practices, physical activity and social determinants of NCDs
**Corresponding Author:**	Sudesh Raj Sharma, PhD Candidate<br>DIYASU Community Development Centre<br>Biratnagar, Morang NEPAL
**Keywords:**	diet; Physical activity; social determinants; non-communicable diseases
**Abstract:**	Unhealthy dietary habit and physical inactivity are major risk factors of non-communicable diseases (NCDs) globally. The objective of this paper was to describe the role of dietary practices and physical activity in the interaction of the social determinants of NCDs in Nepal, a developing economy. The study was a qualitative study design involving two districts in Nepal, whereby data was collected via key informant interviews (n=63) and focus group discussions (n= 12). Thematic analysis of the qualitative data was performed, and a causal loop diagram was built to illustrate the dynamic interactions of the social determinants based on the themes. The study also involved sense-making sessions with policy level and local stakeholders. Four key interacting themes emerged from the study describing current dietary and physical activity practices, influence of junk food, role of health system and socio-economic factors as root causes. While the current dietary and physical activity-related practices within communities were unhealthy, the broader determinants such as socio-economic circumstances and gender further fuelled such practices. The health system has potential to play a more effective role in the prevention of the behavioural and social determinants of NCDs.
**Order of Authors:**	Sudesh Raj Sharma, PhD Candidate
	Anna Matheson
	Danielle Lambrick
	James Faulkner
	David W. Lounsbury
	Abhinav Vaidya
	Rachel Page
**Opposed Reviewers:**	
**Response to Reviewers:**	Response to reviewers<br>1. Please ensure that your manuscript meets PLOS ONE's style requirements, including those for file naming. The PLOS ONE style templates can be found at https://journals.plos.org/plosone/s/file?id=wjVg/PLOSOne_formatting_sample_main_body.pdf and https://journals.plos.org/plosone/s/file?id=ba62/PLOSOne_formatting_sample_title_authors_affiliations.pdf<br>- Thank you for your suggestion. We have reviewed and reformatted the manuscript as per the PLOS ONE style.<br><br>2. We noted in your submission details that a portion of your manuscript may have been presented or published elsewhere. [This article is different from a related published (https://bmcpublichealth.biomedcentral.com/articles/10.1186/s12889-020-09446-2) article in terms of findings and discussion. Since the article is based on my PhD thesis, articles that have been published (or will be published) have similar

Powered by Editorial Manager® and ProduXion Manager® from Aries Systems Corporation

methods section (and hence the use of same figure relating to methodology and study framework).] Please clarify whether this [conference proceeding or publication] was peer-reviewed and formally published. If this work was previously peer-reviewed and published, in the cover letter please provide the reason that this work does not constitute dual publication and should be included in the current manuscript.
- This is the second paper from the PhD thesis and includes results about diet and physical activity. The first paper concentrated on results from the PhD focussing on tobacco and alcohol use (https://bmcpublichealth.biomedcentral.com/articles/10.1186/s12889-020-09446-2) which has been peer-reviewed and published. Since the first published paper and this paper on diet and physical activity are from the first authors PhD thesis, we have used and referenced figures relating to the methods and study framework from the published paper in the submitted paper focusing on diet and physical activity. The introduction, findings and discussion are entirely different to the published paper and have not been published elsewhere and hence, we do not feel this constitutes dual publication.

.
3. PLOS requires an ORCID iD for the corresponding author in Editorial Manager on papers submitted after December 6th, 2016. Please ensure that you have an ORCID iD and that it is validated in Editorial Manager. To do this, go to 'Update my Information' (in the upper left-hand corner of the main menu), and click on the Fetch/Validate link next to the ORCID field. This will take you to the ORCID site and allow you to create a new iD or authenticate a pre-existing iD in Editorial Manager. Please see the following video for instructions on linking an ORCID iD to your Editorial Manager account: https://www.youtube.com/watch?v=_xcclfuvtxQ
- ORCID ID of the corresponding author (SS): https://orcid.org/0000-0002-7880-5517

4. Please review your reference list to ensure that it is complete and correct. If you have cited papers that have been retracted, please include the rationale for doing so in the manuscript text, or remove these references and replace them with relevant current references. Any changes to the reference list should be mentioned in the rebuttal letter that accompanies your revised manuscript. If you need to cite a retracted article, indicate the article's retracted status in the References list and also include a citation and full reference for the retraction notice.

- The reference list has been reviewed and is complete and correct.

[Note: HTML markup is below. Please do not edit.]

Reviewers' comments:

Reviewer's Responses to Questions
Comments to the Author

1. Is the manuscript technically sound, and do the data support the conclusions?

The manuscript must describe a technically sound piece of scientific research with data that supports the conclusions. Experiments must have been conducted rigorously, with appropriate controls, replication, and sample sizes. The conclusions must be drawn appropriately based on the data presented.
Reviewer #1: Partly
Reviewer #2: Yes
Thank you for considering our manuscript as scientifically and technically sound.

____
2. Has the statistical analysis been performed appropriately and rigorously?
Reviewer #1: Yes
Reviewer #2: N/A
We agree that statistical analysis is not applicable for this study.

____
3. Have the authors made all data underlying the findings in their manuscript fully available?

The PLOS Data policy requires authors to make all data underlying the findings described in their manuscript fully available without restriction, with rare exception

	(please refer to the Data Availability Statement in the manuscript PDF file). The data should be provided as part of the manuscript or its supporting information, or deposited to a public repository. For example, in addition to summary statistics, the data points behind means, medians and variance measures should be available. If there are restrictions on publicly sharing data—e.g. participant privacy or use of data from a third party—those must be specified.<br>Reviewer #1: No<br>Reviewer #2: Yes<br><br>We agree with reviewer 1 that still there is limitation in data availability and have shared that with editors. We are in the process of making data available public once we review the transcripts and make sure all personal identifiers of the respondents are removed, as Nepal is a small country and participants from policy level are easily identified. We hope to make the data publicly available by this year.<br>____<br>4. Is the manuscript presented in an intelligible fashion and written in standard English?<br><br>PLOS ONE does not copyedit accepted manuscripts, so the language in submitted articles must be clear, correct, and unambiguous. Any typographical or grammatical errors should be corrected at revision, so please note any specific errors here.<br>Reviewer #1: Yes<br>Reviewer #2: Yes<br>Thank you.<br>____<br>5. Review Comments to the Author<br><br>Please use the space provided to explain your answers to the questions above. You may also include additional comments for the author, including concerns about dual publication, research ethics, or publication ethics. (Please upload your review as an attachment if it exceeds 20,000 characters)<br>Reviewer #1: This is a very interesting and mostly well-written paper. The authors utilize appropriate methods to draw their conclusions<br>Some minor comments<br>1. Please consider using sex instead of gender, given you did not record one's gender. In case you measured one's gender, this should be more clearly presented in the methods<br>-Thank you for your kind suggestion and we agree that we have not measured gender within this paper. However, gender reflects socially constructed characteristics of male and female. We have indeed provided some context such as Nepal being patriarchal society and having more social and economic power as a breadwinner of the family. Also, our findings and discussion are linking those gendered experiences with diet and physical activity related practices. Thus, we feel that it makes more sense if we keep the term “gender” within the manuscript.<br><br>2. The manuscript requires some English editing<br>-The English has been reviewed by co-authors who are native English speakers.<br>3. Avoid using terms such as "causal relationship", "influence" etc. Given your study design cannot support these claims, please replace with association etc<br>-Thank you for your kind suggestion. We have revised and replaced “causal relationship” with “association” accordingly. However, the term “Causal Loop Diagrams” is a standard method/tool name and we have not changed it. Also, as this is a qualitative study, keeping “influence(s)” made more sense (qualitative term) than replacing with “association” (quantitative term) and hence have decided to keep the term “influence (s)” as it is.<br>Reviewer #2: Very timely and useful research. Probably, on of the most critical public health issue of our time is the growing burden of non-communicable disease. Most of these diseases are preventable and heavily dependent on behavior change. Changing people’s behavior requires a thorough understanding of the root causes of behaviors and health system-related structural issue and the interlinkages with social-determinants of health leading to NCDs. The authors clearly described and organized their findings along these lines. Just two question.<br>1. Why were the study areas purposively selected? What was the rational for this judgment sampling rather than random selection? Was it to save time? I understand

	the application of the method to identify participants within the selected communities, as you stated because of their knowledge and experience. But, why were the communities selected through this method?<br>-This is a qualitative study and often, cases are purposively chosen. In the study, the main purpose was to choose different context i.e. urban, semi-urban and rural settings, and was also selected in consultation with District Health Offices. Time and convenience of the first author (principal investigator) also influenced the choice of districts which is acceptable in purposive sampling. In qualitative studies, often random sampling is not the choice of sampling.<br><br>2. Most of the findings do not seem new. It would be helpful, if the authors emphasized on what is the key new knowledge versus what was already known. For example, we know urbanization, increasing availability and consumption of junk food, alcohol consumption, smoking and reduced physical activities are risk factors for NCDs. Is the key on the findings the Nepalese context?<br>-The findings are especially important in Nepalese context where the focus is on changing behaviour. Yes, the findings may not be new for high-income countries but, for low income countries like Nepal, the authors hope that this paper can provide a whole new way of seeing a public health issue, taking a systems perspective and designing appropriate actions. This paper provides qualitative evidence on social determinants of dietary and physical activity practices which is very scarce in Nepalese (or low income country) and we hope that this provides some systemic insights for actions by concerned health agencies in Nepal.<br>____<br>6. PLOS authors have the option to publish the peer review history of their article (what does this mean?). If published, this will include your full peer review and any attached files.<br><br>If you choose “no”, your identity will remain anonymous but your review may still be made public.<br><br>Do you want your identity to be public for this peer review? For information about this choice, including consent withdrawal, please see our Privacy Policy.<br>Reviewer #1: No<br>Reviewer #2: No<br><br>[NOTE: If reviewer comments were submitted as an attachment file, they will be attached to this email and accessible via the submission site. Please log into your account, locate the manuscript record, and check for the action link "View Attachments". If this link does not appear, there are no attachment files.]<br><br>While revising your submission, please upload your figure files to the Preflight Analysis and Conversion Engine (PACE) digital diagnostic tool, https://pacev2.apexcovantage.com/. PACE helps ensure that figures meet PLOS requirements. To use PACE, you must first register as a user. Registration is free. Then, login and navigate to the UPLOAD tab, where you will find detailed instructions on how to use the tool. If you encounter any issues or have any questions when using PACE, please email PLOS at figures@plos.org. Please note that Supporting Information files do not need this step.<br>____<br>In compliance with data protection regulations, you may request that we remove your personal registration details at any time. (Remove my information/details). Please contact the publication office if you have any questions.
**Additional Information:**	
**Question**	**Response**
**Financial Disclosure**<br><br>Enter a financial disclosure statement that	SRS studied towards his PhD at Massey University supported by Massey University Doctoral Scholarship and Massey University Graduate Research Support. This paper is part of the doctoral study. The funding support has no role in the study design, data

describes the sources of funding for the work included in this submission. Review the submission guidelines for detailed requirements. View published research articles from *PLOS ONE* for specific examples.<br><br>This statement is required for submission and **will appear in the published article** if the submission is accepted. Please make sure it is accurate.<br><br>**Unfunded studies**<br>Enter: *The author(s) received no specific funding for this work.*<br><br>**Funded studies**<br>Enter a statement with the following details:<br>• Initials of the authors who received each award<br>• Grant numbers awarded to each author<br>• The full name of each funder<br>• URL of each funder website<br>• Did the sponsors or funders play any role in the study design, data collection and analysis, decision to publish, or preparation of the manuscript?<br>• **NO** - Include this sentence at the end of your statement: *The funders had no role in study design, data collection and analysis, decision to publish, or preparation of the manuscript.*<br>• **YES** - Specify the role(s) played.<br><br>* typeset	collection and analysis and preparation and publication decisions relating to the study. There is no any other external funding to report.
**Competing Interests**<br><br>Use the instructions below to enter a competing interest statement for this submission. On behalf of all authors, disclose any competing interests that could be perceived to bias this work—acknowledging all financial support and any other relevant financial or non-financial competing interests.<br><br>This statement is required for submission and **will appear in the published article** if the submission is accepted. Please make sure it is accurate and that any funding	We declare no conflict of interest.

sources listed in your Funding Information later in the submission form are also declared in your Financial Disclosure statement.<br><br>View published research articles from *PLOS ONE* for specific examples.<br><br>**NO authors have competing interests**<br><br>Enter: *The authors have declared that no competing interests exist.*<br><br>**Authors with competing interests**<br><br>Enter competing interest details beginning with this statement:<br><br>*I have read the journal's policy and the authors of this manuscript have the following competing interests: [insert competing interests here]*<br><br>* typeset	
**Ethics Statement**<br><br>Enter an ethics statement for this submission. This statement is required if the study involved:<br><br>• Human participants<br>• Human specimens or tissue<br>• Vertebrate animals or cephalopods<br>• Vertebrate embryos or tissues<br>• Field research<br><br>Write "N/A" if the submission does not require an ethics statement.<br><br>General guidance is provided below. Consult the submission guidelines for detailed instructions. **Make sure that all information entered here is included in the Methods section of the manuscript.**	Formal ethical approvals were taken from the Massey University Human Ethics Committee (SOA 16/37) and Nepal Health Research Council Ethics Committee (Reg. no. 163/2016) respectively. All participants, and in particular disadvantaged and less literate groups, were clearly informed about the purpose of the study. All participants were provided with an information sheet, and a voluntary written consent was obtained from all participants of semi-structured interviews and focus group discussions.

**Format for specific study types**<br><br>**Human Subject Research (involving human participants and/or tissue)**<br>• Give the name of the institutional review board or ethics committee that approved the study<br>• Include the approval number and/or a statement indicating approval of this research<br>• Indicate the form of consent obtained (written/oral) or the reason that consent was not obtained (e.g. the data were analyzed anonymously)<br><br>**Animal Research (involving vertebrate animals, embryos or tissues)**<br>• Provide the name of the Institutional Animal Care and Use Committee (IACUC) or other relevant ethics board that reviewed the study protocol, and indicate whether they approved this research or granted a formal waiver of ethical approval<br>• Include an approval number if one was obtained<br>• If the study involved *non-human primates*, add *additional details* about animal welfare and steps taken to ameliorate suffering<br>• If anesthesia, euthanasia, or any kind of animal sacrifice is part of the study, include briefly which substances and/or methods were applied<br><br>**Field Research**<br><br>Include the following details if this study involves the collection of plant, animal, or other materials from a natural setting:<br>• Field permit number<br>• Name of the institution or relevant body that granted permission	
**Data Availability**<br><br>Authors are required to make all data underlying the findings described fully available, without restriction, and from the time of publication. PLOS allows rare exceptions to address legal and ethical concerns. See the PLOS Data Policy and FAQ for detailed information.	No - some restrictions will apply

Powered by Editorial Manager® and ProduXion Manager® from Aries Systems Corporation

A Data Availability Statement describing where the data can be found is required at submission. Your answers to this question constitute the Data Availability Statement and **will be published in the article**, if accepted.<br><br>**Important:** Stating 'data available on request from the author' is not sufficient. If your data are only available upon request, select 'No' for the first question and explain your exceptional situation in the text box.<br><br>Do the authors confirm that all data underlying the findings described in their manuscript are fully available without restriction?	
**Describe where the data may be found in full sentences. If you are copying our sample text, replace any instances of XXX with the appropriate details.**<br><br>• If the data are **held or will be held in a public repository**, include URLs, accession numbers or DOIs. If this information will only be available after acceptance, indicate this by ticking the box below. For example: *All XXX files are available from the XXX database (accession number(s) XXX, XXX.).*<br>• If the data are all contained **within the manuscript and/or Supporting Information files**, enter the following: *All relevant data are within the manuscript and its Supporting Information files.*<br>• If neither of these applies but you are able to provide **details of access elsewhere**, with or without limitations, please do so. For example:<br><br>*Data cannot be shared publicly because of [XXX]. Data are available from the XXX Institutional Data Access / Ethics Committee (contact via XXX) for researchers who meet the criteria for access to confidential data.*<br><br>*The data underlying the results presented in the study are available from (include the name of the third party*	Transcripts (without any personal identifier and of those who consented to sharing of the interview transcripts) are available on request (Email: yoursudesh@gmail.com; r.a.page@massey.ac.nz) after signing confidentiality agreement due to ethical reasons.

and contact information or URL).<br>• This text is appropriate if the data are owned by a third party and authors do not have permission to share the data.<br><br>* typeset	
Additional data availability information:	

13 May, 2022

The Chief Editor

Plos One

Subject: Submission of revised manuscript (Research article)

Dear madam/sir,

First of all, I would like to sincerely apologize for delayed submission of revision.

I, as a corresponding author of the research article titled '**Dietary practices, physical activity and social determinants of non-communicable diseases in Nepal: a systemic analysis'**, am submitting the revised manuscript for your kind consideration.

In the article, we have highlighted the NCDs problem from systems perspective focusing on the role of two key behavioral risks factors i.e. dietary practices and physical activity. The article explores the complex causal mechanism of NCDs mediated by dietary practices and physical activity in Nepal through case analysis and causal loop diagrams which to our knowledge is first such research in the context of Nepal. We have addressed most of the comments from the peer reviewers and also explain the data availability related ethical issues.

Kindly consider the revised manuscript for publication. We declare no conflict of Interest. We accept all the terms and conditions of the journal to the best of my knowledge.

Kind regards

Sudesh Raj Sharma

DIYASU Community Development Centre, Nepal

PhD, Massey University, New Zealand

Manuscript

Click here to access/download;Manuscript;220406 D_P Manuscript PLos One_Clean.docx

Dietary practices, physical activity and social determinants of non-communicable diseases in Nepal: a systemic analysis

Sudesh Sharma[1,2]*, Anna Matheson[3], Danielle Lambrick[4], James Faulkner[5], David W. Lounsbury[6], Abhinav Vaidya[7], Rachel Page[2]

[1] DIYASU Community Development Centre, Biratnagar, Morang, Nepal

[2] Massey University, Wellington, Wellington Region, New Zealand

[3] Victoria University of Wellington, Wellington, Wellington Region, New Zealand

[4] University of Southampton, Southampton, Hampshire, UK

[5] University of Winchester, Winchester, Hampshire, UK

[6] Albert Einstein College of Medicine, Bronx, New York, US

[7] Kathmandu Medical College, Kathmandu, Bagmati, Nepal

*Corresponding author

Email: yoursudesh@gmail.com (SS)

Abstract

Unhealthy dietary habit and physical inactivity are major risk factors of non-communicable diseases (NCDs) globally. The objective of this paper was to describe the role of dietary practices and physical activity in the interaction of the social determinants of NCDs in Nepal, a developing economy. The study was a qualitative study design involving two districts in Nepal, whereby data was collected via key informant interviews (n=63) and focus group discussions (n= 12). Thematic analysis of the qualitative data was performed, and a causal loop diagram was built to illustrate the dynamic interactions of the social determinants based on the themes. The study also involved sense-making sessions with policy level and local stakeholders. Four key interacting themes emerged from the study describing current dietary and physical activity practices, influence of junk food, role of health system and socio-economic factors as root causes. While the current dietary and physical activity-related practices within communities were unhealthy, the broader determinants such as socio-economic circumstances and gender further fuelled such practices. The health system has potential to play a more effective role in the prevention of the behavioural and social determinants of NCDs.

Background

Unhealthy dietary habit and physical inactivity have been identified as key risk factors in increasing non-communicable diseases (NCDs) in developing countries [1, 2]. It has been estimated that dietary and physical activity risks attribute to approximately 5.3 million premature deaths annually [3]. In Nepal, the last Stepwise approach to surveillance (STEPS) survey has shown that about 99% of people did not consume sufficient fruits and vegetables but only 3% did ~~not~~ have adequate physical activity [4]. However, the problem of physical inactivity was more pervasive in the urban and peri-urban areas with more than 5% of urban respondents categorised as physically inactive [4]. A recent study showed that inadequate physical activity among adolescents was 85%, ~~indicating the shift towards physical inactivity in Nepal~~ [5].

Increasingly, there is evidence relating to socio-economic and environmental determinants influencing diet and physical activity, often collectively termed social determinants [6, 7]. Global health agencies and experts have been pushing the agenda of NCDs prevention in both developed and developing nations through focusing on the social determinants of health [8-12]. In 2008, the World Health Organization proposed the landmark *Social Determinants of Health Framework*, which highlighted the association of proximal and distal determinants of health and suggested directions for actions [11]. Since then, there has been increasing focus in understanding and taking action on the social determinants of complex health issues such as NCDs globally [13]. However, developing countries are far behind in their understanding and actions on the social determinants of health compared to developed countries [13-17]. On a positive note, some Latin American and Caribbean countries have initiated significant steps in addressing the social determinants of NCDs [18, 19].

Global evidence has suggested that physical inactivity as well as junk food consumption is rapidly increasing due to social, environmental and economic influences [6, 7, 20]. Rapid urbanisation and changing nature of work (sedentary nature and increasing mechanisation of physical work) are impacting physical activity and dietary patterns [6, 21]. A Lancet review has suggested that physical inactivity is increasing among lower socio-economic groups [21]. Moodie et. al. suggested that transnational companies are driving the junk food epidemic globally, resulting in increasing NCDs, even in developing countries [20]. Studies from Nepal have shown that urban population have an increased risk of chronic diseases due to

ready access to junk food and limited avenues for physical activity [4, 22-24]. Further, limited policy and multi-sectoral action on healthy diet and optimal physical activity promotion have resulted in increase in NCDs and their risks [2, 25]. However, there is limited evidence or analysis of the links between social determinants and NCDs mediated by diet and physical activity in Nepalese and developing countries context. This paper assesses the role of dietary practices and physical activity in the interaction of the social determinants of NCDs in Nepal.

Methods

The study design was qualitative study based on systemic intervention (SI) methodology (Fig 1) [26, 27]. SI adopts the critical systems thinking approach and stresses on using multiple methods to understand the complex problems. A combination of case study and system dynamics (causal loop diagram) methods, along with participatory sense-making workshops, were adopted within the research design.

Fig 1. Systemic intervention study design to study social determinants of NCDs in Nepal (Image republished from authors' open access article [27])

Study area and study participants

The study was conducted in Nepal from July until October 2016. Two districts (Bhaktapur and Morang) from Nepal were purposively selected as case districts. Bhaktapur is a hilly district near the capital city of Nepal and has a sub-tropical cold and humid climate. Morang is a plain district located in the eastern part of Nepal and has a sub-tropical hot and humid climate. Within each case district, one municipality and two village development committees (VDC) were selected for community level data collection.

Community members affected by NCDs and key stakeholders from community, district and policy level were the participants of the study. The key stakeholders were purposively selected based on their current engagement (or potential engagement) in NCDs prevention and health promotion in the case districts and at policy level in Nepal. They included members of *NCDs Multi-sectoral Action Plan Steering Committee*, academia, non-government organisations, District Health Officer, Local Development Officer, community-based organisations, local health workers and village leaders. The communities for focus

group discussion (FGD) were identified through the referral of respective district health offices and local health institutions.

Data collection

Data collection involved semi-structured interviews with key stakeholders from the case districts and policy level. Policy level data were collected to supplement the information from case districts. Semi-structured interview guideline so developed were guided by the adapted social determinants of health framework (Fig 2).

Fig 2. Adapted Social Determinants of Health Framework for the study of the social determinants of NCDs in Nepal (Image republished from authors' open access article [27])

The sampling strategy for the study entailed identifying and participating individuals knowledgeable and experienced in the issue of interest i.e. NCDs prevention at case district and policy level in Nepal. The purpose of the purposive sampling was to capture the different perspectives and contexts on the NCDs prevention issue guided by the study framework (Fig 2). Purposive sampling is often used in qualitative studies for identifying and interviewing potential information-rich subjects on a complex issue like NCDs [28]. *Maximum variation* strategy was adopted to ensure diverse perspectives across the health and social sectors in Nepal were captured relating to complex issue of NCDs [28]. Thirty-nine participants were interviewed from the two case study sites (Bhaktapur and Morang) and 24 participants from policy level. All of the interviews were carried out by the first author in Nepali language. The time of interview ranged from 30 minutes to one hour.

Further, 12 focused group discussions (FGDs) were conducted within the selected municipalities and VDCs of the case districts. Each FGD included five to 10 community people experiencing and/or caring for family members with NCDs and their metabolic risks. The purpose of FGDs was to capture negotiated views on NCDs and risks as experienced by individuals, families and community members belonging to different socio-economic groups. Therefore, in each VDC/municipality, two FGDs were conducted with one group representing a socio-economically disadvantaged community and the other a more advantaged/mixed community. The FGDs were facilitated by the first author with the help of local volunteers. The duration of FGDs ranged from 45 minutes to one hour.

Data management and analysis

The interviews and FGDs audio recordings were first transcribed in Nepali and then translated them into English for coding and thematic analysis. A *Framework Approach* was used to undertake the thematic analysis guided by the study framework (Fig 2) [29]. Themes were identified, grouped and coded by using both a deductive approach based on the study framework as well as more inductive approaches to allow for emergent themes. Case analysis helped in framing the qualitative and quantitative data for the local context and iterating on association of various social determinants. A systems map or causal Loop Diagram (CLD) was developed to illustrate the association of the social determinants of NCDs indicated by thematic analysis. Causal loop diagramming is a system dynamics technique to build a system map of a problem of interest that illustrates the causal association through feedback structures that are loopy in nature [30]. CLD consists of two types of feedback loops: balancing and reinforcing loops, and these loops have variables connected by uni-directional arrows with either positive or negative signs on them. A positive sign indicates that if the independent variable increases or decreases, the dependent variable also increases or decreases in the same direction. A negative sign indicates the exact opposite meaning i.e. if independent variable increases, then the dependent variable decreases and vice versa. The balancing loop is a goal-seeking loop, which is indicated by *B* within CLD and indicates the stabilising feature of the loop. In general, causal loops with an intervention are of a balancing nature that tried to mitigate the problem. On the other hand, reinforcing loops (indicated by *R* within CLD) depict a mechanism that produces a result that influences more of the same action, thus resulting in either growth or decline. An example of reinforcing loop can be the vicious cycle of poverty and ill health. *Dedoose (Socio-cultural Research Consultants)* and *MS Excel 2016 (Microsoft)* [31] were used to manage the qualitative data [32] and *Vensim (Ventana Systems Inc.)* was used to build CLD [33].

Stakeholder Validation

Stakeholder validation was conducted through organising three sense-making workshops, two at respective case districts and one at national level during January/February, 2018. The workshops helped to further enrich the analysis through the feedback and suggestions from the stakeholders. These workshops, indeed, became opportunities to sensitise the stakeholders about the impending epidemic in Nepal. The workshops proved useful in reaching the purpose of this research and the methodological stance of systemic intervention methodology.

Ethical Consideration

Formal ethical approvals were taken from the Massey University Human Ethics Committee (SOA 16/37) and Nepal Health Research Council Ethics Committee (Reg. no. 163/2016) respectively. All participants, and in particular disadvantaged and less literate groups, were clearly informed about the purpose of the study. All participants were provided with an information sheet, and a voluntary written consent was obtained from all participants of semi-structured interviews and focus group discussions.

Findings

Four key interacting thematic areas relating to dietary practices and physical activity emerged, which were utilised to develop a causal loop diagram and demonstrate dynamic complexity.

Theme 1: Practice relating to diet and physical activity contributing to increased risk

Key informants and focus group participants indicated that current food habits among most Nepalese were not balanced, which was potentially leading to the problem of NCDs. A focus group participant from rural Morang shared:

> *"Nepalese food is taken three times a day. And there is rice every time, even in the snack. That is why now, 2 out of 4 people of a family have high sugar level."* (ID: 69)

Some participants expressed pre-existing and predominant beliefs, which was sustaining such unhealthy dietary practices.

> *"We eat enough food (at night). Since that has to withstand us up to 10 am in the morning."* (ID: 69; Rural Morang; FGD Participant)

One key observation highlighted by participants from the case districts was that the traditional practice of healthy eating was being gradually displaced by westernised practice of eating, which is unhealthy for the Nepalese context. A FGD participant from rural Bhaktapur lamented:

> *"We used to have "Dhido" in the morning and in the evening. We used to run home from school during lunchtime, have the lunch and return back to school. But these days vehicles come to pick up and drop our children and there is no time to prepare lunch for the children. So junk foods are easy."* (ID: 76)

A policy level key informant explained that overwhelming availability and appeal of junk foods were affecting local dietary practices.

> *"In case of food habit, our raw food/fruits habits have been changed to junk food based diet. Junk food are readily available, easy to cook and do not need much effort. There is an increase in the use of junk food from a very early age." (ID: 3; Policy Stakeholder; Health)*

Some participants stressed that the Nepalese culture and traditions have potential to promote physical activity and healthy diet but they continue to be ignored. Offering prayers to different temples in a single walk (Newari culture), yoga and meditation (Hindu culture), *Namaz* prayer (ritual Muslim prayer, which also involves different body movements) were stressed by participants as practices that have physical activity significance.

> *"In context of our geographically and culturally diverse country, we have our own traditions of practicing yoga, meditations, bipasyana etc. but these aren't practiced in our daily schedule." (ID: 12; Policy Stakeholder; Health)*

Participants also reported the decline in physical activities like walking due to increasing use of motor vehicles in the communities.

> *"People who used to walk now use bicycle and motorbike now and that makes easy for them. Because of that too, it has been decreased now." (ID: 66; Urban Morang; FGD Participant)*

A case study district stakeholder elaborated that people often resort to physical activity practice, including yoga and morning walks, after they start suffering from diseases or metabolic risk.

> *"If we ask 100 people who come to Yoga if they do not have any such disease or conditions, only few hands would raise up." (ID: 30; Bhaktapur District Stakeholder; Non-health)*

Theme 2: Health sector issues delaying actions to improve dietary practice and physical activity

Key policy stakeholders shared that health sector has historically considered NCDs as a curative care agenda instead of a multi-sectoral prevention agenda. This has led to current

lack of concrete structure and leadership relating to NCDs prevention which is creating a delay in coordinated action for nutrition and physical activity promotion.

> *"Our health system is resisting to adapt to addressing NCDs issues from health promotion and prevention aspect." (ID: 18; Policy Stakeholder; Health)*

As a result, district stakeholders reported that the district health system and below have limited NCDs prevention-related activities in addition to systemic health system issues. Health workers lacked training in NCDs prevention and the office had limited resources at their disposal. A district stakeholder shared how the current health system was more focused on curative care than preventative action.

> *"Our [Primary Health Care] network is very good. But, it is less focus on primary prevention compared to curative. Our system can do more primary prevention." (ID; 25; Bhaktapur District Stakeholder; Health)*

A key policy stakeholders expressed limited health sector intervention in curbing marketing and promotion of junk food leading to current junk food based dietary practice and displacing the traditional diet.

> *"Second one is food habit where major issue is aggressive advertisement of junk foods. What is happening is, gradually we are leaving our indigenous food habits and shifting towards the trend of consumption of junk foods may be as a fashion or may be as a status symbol." (ID: 9; Policy Stakeholder; Non-health)*

A rural health worker shared how the dietary practice has shifted in the last 10 years as follows:

> *"Nowadays people are eating lot of fast food. Ten years earlier people used to have home beaten rice and beans; corn (pop-corn) and would have a balance diet. But now people do not eat at home; they eat outside and mostly junk food." (ID: 35; Rural Bhaktapur; Health Worker)*

In addition, participants stressed that increased motorisation and lack of systemic infrastructure were leading to limited physical activity in the case studies. There has been a rapid development in road infrastructure and motorisation of public and private transport systems. However, due to lack of awareness regarding active travel and luxury-seeking behaviour, this has been contributing to physical inactiveness to some extent.

"These days, vehicles are available right in our doorsteps. Now instead of walking to the place that takes 30 minutes, we wait an hour for the vehicles to take us there." (ID: 65; Urban Morang; FGD Participants)

Urban areas of Nepal have poor urban planning, leading to limited green and open spaces for promoting physical activity among the residents. Though there are policies and regulations relating to urban and housing design in place, residents simply ignore them, and the regulations are not strongly implemented enough to have any meaningful impact. This is leading to cumulative effect of limited space as explained by a policy stakeholder:

"What has happened is lack of open space has become an increasing problem because of population growth and exposure to open space is diminishing /decreasing. This leads to decrease in people's habit of roaming, walking, exercise and lack of good/high oxygen concentration. This trend is slowly fading away even in rural areas and condition has worsened in urban areas." (ID: 11; Policy Stakeholder; Health)

Some participants linked limited physical activity with system ignorance and limited promotion of yoga and traditional physical activity. Often the system was not focusing on instilling habits of physical activities among the young people.

"We ourselves are responsible for the current state of yoga. We couldn't direct the young generations to a correct track." (ID: 52; District Stakeholder Morang; Non-health)

Theme 3: Gendered-related norms and cultural shifts affecting physical activity and dietary practice

There were some revelations about the influence of gender on the physical activity and dietary practice. Participants shared that in urban areas, females tended to be physically inactive and overweight/obese.

"In urban and semi urban areas obesity is seen more in married female." (ID: 5; Policy level)

However, there were some positive actions being initiated within urban communities where more females compared to male were engaged in yoga.

> *"You may see that many females come to the yoga and are often married. Not many males come; probably due to complacency and laziness." (ID: 44; Urban Bhaktapur; Social Worker)*

Similarly, participants shared that more and more families of foreign employed males were migrating to urban centres for a better life. As a result, female members within such families were detached from their regular agricultural works in the rural context and confined to limited space rented houses, which significantly reduced their physical activity.

> *"If we talk about those families whose members are working abroad, their families now have started living in the market areas and depend upon market for every necessities including the vegetables which once they themselves used to grow." (ID: 7; Policy Stakeholder; Health)*

In rural areas of case districts, women who used to be engaged in agricultural activities and animal rearing have rapidly given up such practices.

> *"In our time we used to run to the field soon after having the meal. We used to cultivate different crops and plants like maize, mustard and many more. We used to grow mustard here like in Chitwan but now there are only bushes and grasses in those lands." (ID: 76; Rural Bhaktapur; Female FGD Participant)*

Theme 4: Migration affecting agriculture sector and exposing vulnerable families to obesogenic environment

The participants pointed that migratory movement of youths and adults and their families from rural to urban and foreign countries was one of the key reasons for rapid decline of agriculture activities. The migration created a shortage of agricultural workforce in rural areas. The migration was mainly driven by limited economic opportunities in rural areas and prospect of better opportunities in urban and foreign land. A policy stakeholder noted that foreign migration was having double whammy effects in terms of affecting the agriculture sector as well as exposing vulnerable families to unhealthy lifestyle.

> *"At least one member of a household is earning in foreign land. Now the proportion of population involved in agriculture has decreased in recent times. People's profession has changed. Along with the change in profession, people's lifestyle has also changed." (Policy Stakeholder; ID: 6; Health)*

A rural stakeholder further added that the decline in agricultural activities was directly resulting in increased dependency on commercially available food and decrease in physical activity in rural areas.

> *"Importantly, agricultural activity is on a decline, everyone is now buying and eating food items in rural areas. People do not engage in hard agricultural practices as they used to do. People now eat too much of unhealthy products." (ID: 55; Rural Morang; Health Facility Management Committee member)*

One of the key stakeholders explained that despite unhealthy dietary practice and physical inactivity being widespread, vulnerable group were disproportionately affected in terms of health effect.

> *"There are certain things about poor population as they have unhealthy diet but they are involved in lots of physical activity during digging, working; the disease differs between the poor and the rich. Physical activity is the good aspect among the poor but there are many other factors that are negative like unhealthy diet (consume more fast foods), inaccessibility of treatment facility and many stress factors among the poor people results in their diseased condition. Beside that behaviour of smoking and alcohol consumption among poor is another cause of disease." (Policy Stakeholder; ID: 4; Health)*

CLD of major sub-systems depicting the interaction of the social determinants of unhealthy diet and physical inactivity and NCDs

The CLDs below have been derived from the thematic analysis and show the dynamic interaction of the key variables discussed above contributing to the problem of NCDs in Nepal. As indicated, the development of the CLDs was an iterative process and was conducted simultaneously with thematic analysis. The CLDs depicts three prominent feedback mechanisms or sub-systems displaying the interactions of the social determinants of NCDs: prevention delay, demand-supply and socio-economic influence.

In Fig 3, the prevention delay sub-system consists of delayed balancing loops, which highlight themes that indicate limited and delayed action by the health system towards addressing unhealthy diet and physical inactivity-related practices as indicated in the results. Awareness-raising campaigns and multi-sectoral coordination for promoting sustainable food systems and physical activity-friendly environments were lacking for prevention of NCDs.

The health system was possibly guided by the mental model that unhealthy diet and physical inactivity were more due to individual behaviour, hence the interventions in developing countries were limited to campaigns and medical services provision. The CLD highlighted the themes indicating that multi-sectoral coordination for sustainable food systems and improving physical infrastructure were continually neglected by the overall health and social system. The themes also indicated that junk food companies are using various tactics to promote their products as a healthier option. The same sub-system hints at the curative orientation of the health system (i.e. the increasing chronic diseases problem in turn put pressure on the government to provide curative care services, leaving limited resources for preventative actions, including multi-sectoral coordination).

Fig 3. Prevention delay sub-system showing the interaction of governmental response to promote healthy diet and physical activity, other sub-systems and NCDs

Demand-supply sub-system (Fig 4) highlights how individuals and communities were being framed for consuming junk food and drinks due to limited policy and programmatic interventions relating to junk food/drink. The demand-supply sub-system mainly comprised of reinforcing loops and showed that junk food availability was resulting in increased consumption and affecting local food systems as well as social practices in the case district of Nepal. The extensive marketing of such products as healthy, easy to prepare, cost-effective, family food, etc. have driven the consumption of junk food. The junk food companies are possibly utilising their economic strength to influence both government and public through policy influences and marketing as indicated by study participants.

Fig 4. Demand-supply sub-system showing the interaction of availability and sales of junk food, other sub-systems and NCDs

Socio-economic sub-system (Fig 5) can be conceptualised to distally drive the other two sub-systems towards increasing the burden of NCDs as per the thematic analysis. The sub-system shows the dynamic complexity about how junk foods, physical inactivity and social and economic influences were interacting, which was giving rise to NCDs problems. In particular, socio-economic situation was fuelling the migration of vulnerable groups from rural to city areas and foreign countries, which was not only pushing the rural agriculture system to the verge of collapse but also exposing rural people to unhealthy lifestyles. The

sub-system also shows the disproportionate vulnerability and health effects experienced by the female members of migrant families and female in general.

Fig 5. Socio-economic influence sub-system showing the interaction of socio-economic status, other sub-systems for dietary and physical activity practices and NCDs

Discussion

The causal association of the diet and physical activity with NCDs was complex and is discussed here in the light of existing evidence and from a systems perspective. Specifically, we utilise system archetypes (Fig 6), a simpler version of CLDs, to present the mechanism in a way that generates insights for health system action [34].

Fig 6 System archetypes showing different mechanisms of influence of dietary and physical activity practices on NCDs

Promotion of healthy dietary behaviour and physical activity

In this study, prevention-delay sub-system indicated that the health system has not been structured and functioning effectively to promote healthy behaviours relating to diet and physical activity. The health system in many developing countries like Nepal is yet to prioritise the prevention of NCDs and their behavioural risks [35-38]. In Nepal, evidence indicates that NCDs have been considered as a curative service agenda with increasing budgetary provisions in hospitals, patient care and development of clinical human resources rather than prevention [39]. This resonates with the *Fixes that Fail* system archetype (Fig 6A).

Further, in many developing countries, national strategies for improving diet and physical activity exist [40-43] but most strategies are not effectively implemented due to limited resources and system inefficiency [44, 45]. In particular, developing countries are often lagging behind in terms of formulating and implementing legislations and programmes relating to control of junk food as well as developing physical activity-friendly infrastructures [20, 46], similar to the findings in this study. While developed countries are gradually addressing the junk food epidemic through policy measures, developing countries do not have any mechanism in place to control the junk food epidemic. The junk food companies are

being increasingly compared to tobacco/alcohol companies due to their marketing strategies that target vulnerable children and population groups and their role in affecting the local food systems and ultimately health [20]. Further, poor systematic infrastructure-related policy action has been contributing to inadequate physical activity around the world [46, 47]. Prevention delay sub-system has illustrated that disease prevention and health promotion efforts in Nepal have been offset by systemic issues within health system. As a result, complex problems like NCDs continue to persist and there is tendency of health system to focus on easily implementable solutions than a comprehensive approach which require health system strengthening. This can be captured by the *Shifting Burden* archetype (Fig 6B). Many developing countries face similar health system challenges such as poor governance and management, resulting in inefficiency and ineffectiveness [48-51]. The health system continues to be ineffective and inefficient due to top-down approach, and leadership and management issues. Public health actions in developing countries are often short-sighted and implemented without collaborating effectively with other sectors. Strong leadership, appropriate system structure and political commitment could play a significant role in strengthening the health system and supporting comprehensive prevention initiatives in developing countries like Nepal [45, 52]. This shift will ensure more time and resources for the implementation of the prevention initiatives that is essential for combating complex problems like NCDs and their behavioural risks [53, 54].

Junk food access and marketing influencing consumption of junk foods

Demand-supply subsystem showed an increased access to highly processed food by everyone, especially young people. Evidence suggests that the junk food habit is very pervasive among children and adults in both urban and rural areas of South Asia region [55-58]. Further, a key mechanism that was prominent in the findings was the deskilling of the traditional food preparation due to wider availability of the junk food and fast food. Evidence from around the world has also confirmed that traditional-based diets are gradually being replaced by junk food habit [59-62]. A review has highlighted how the local food systems were being shaped more by profit motives of the companies than by optimal nutrition [63]. In addition, migration of male youths, especially from rural areas, was driving the migration of families to urban areas, which is suddenly exposing families to an obesogenic environment.

Marketing of junk food is another key reason for the increasing access of the junk food as shown by different types of evidence [64] and highlighted by demand-supply sub-system.

Often, the junk food companies through quality advertisements would associate their products with some social services or claim their products to be nutritious and can be enjoyed by an entire family [65]. Such practices of social marketing and misleading vulnerable population has been well documented in developed and developing countries [65-67]. Similar to the findings of this study, a report identified the price factor, easy availability and lack of government control were some of the factors that were leading to families providing children with junk food [65].

Enforcement agencies have limited ability to control junk food due to lack of regulations and positive framing of junk food by companies producing junk food. Junk food and sugar-sweetened beverages issues have not received much attention in public health policies and action in developing countries [20], which has also been reflected in the themes of this paper. Further, the effects of junk foods on local food systems have not been explored extensively. Developed countries with ample evidence of the effects of junk food are still facing stiff challenges in preventing the junk food induced obesity epidemic [68, 69]. The labelling of products is still a contested issue in Australia and New Zealand [70]. A Lancet review suggested that tactics of food companies influencing national policies can be considered similar to that of tobacco companies [20]. The *Drifting Goal* system archetype depicts the influence of food industries in delaying public health regulations for junk food such as sugar tax (Fig 6C).

State of physical activity including traditional exercises

As noted in the themes and indicated in the CLD (interaction between all three subsystems), physical activity was on a decline in both urban and rural areas. A study in India showed that physical activity was gradually decreasing in rural India as well due to increasing urban influences, including adopting urban lifestyle in rural settings, motor vehicle access and mechanisation of agricultural activities in rural areas [71]. Research in rural India and Bangladesh show a similar increasing phenomenon of physical inactivity in rural areas [72-74]. This study showed a shift in social practices (such as travelling, working and exercise practices) resulting in adults being physically inactive, which was further complicated by habit of consuming energy-dense food. A similar shift in diet and physical activity has been observed elsewhere in developing countries contributing to NCDs [75-77]. People only resorted to healthy diet and exercise after being affected with metabolic risk, a tendency that can be explained by behavioural and psychological theories [78, 79]. Evidence from South

Asia region evidence has even suggested that people do not readily change their lifestyle even after being affected with NCDs metabolic risks [80].

The study findings suggested that traditional exercises were being ignored, which have potential to keep the general population healthy, based on cultural values and beliefs. The Vedic culture of Nepal (relating to Hindu religion) is rich in such exercise practices, particularly in yoga and meditations. Yoga is becoming increasingly popular in the Western countries due to its benefits [81] but continues to be neglected in Nepal. This can be explained by *Shifting the Burden* archetype (Fig 6D) where the health system has not realised the most effective and preventive measures are based on local socio-cultural practices.

Socio-economic situation, migration and gender effect on dietary practice and physical activity

This study showed that socio-economic factors was influencing the diet and physical activity practices in multiple ways within the case districts. In rural areas, there was limited awareness about diet and physical activity. A relatively higher level of physical activity in rural areas could be attributed to agriculture and farming practices, while in urban areas, despite awareness, practice of balanced diet and physical activity was lacking. Available evidence also indicated this complex relationship [21, 82]. Some evidence showed that higher income groups were physically inactive while in some context, lower income groups had less physical activity [83, 84]. A Lancet article noted this complexity and indicated that there may be a social patterning, which was shifting in the developing economy [21]. Such a shift of social patterning needs to be further researched. In this study, health was secondary to the vulnerable group who were experiencing economic hardship. More and more people are migrating to urban areas due to economic hardship and unequal development, which is exposing people to urban influences of junk food diet and limited physical activity. A study in the US has shown that low-income households have a fatalistic attitude towards their health, possibly due to socio-economic circumstances and lack of ability to control their lives [85].

This study also found an increasing issue of unhealthy diet and physical inactivity among women, both in urban and rural areas. While evidence to support our findings from rural areas was limited, but evidence suggest women in urban areas are more vulnerable to the obesogenic environment than male [57]. Urban females' lack of exercise could be understood

by the fact that they remain mostly inactive due to confinement to household duties and child rearing, an outcome of Nepalese patriarchal social construct. A sense of the low power of females can be interpreted by limited access, time and autonomy for females to care for their health and engage in active lifestyles. Similar findings of limited autonomy among women relating to diet and physical activity related decisions have been reported elsewhere [86]. This study, particularly, identified the increased health vulnerability of rural women who migrated to urban areas due to sudden exposed to urban lifestyle. A more critical observation is needed to understand the level of autonomy and access to services of the women among different socio-economic group in both rural and urban areas.

The study findings indicated that the rapid migration and resulting decline in rural agricultural activity had both health and economic implications. The migration of economically active youths and their families to cities and foreign countries not only affected their lifestyle but also affect the local food systems and economy. A review has suggested that locally grown fresh foods were being replaced by imported vegetables and international junk food and supplies, which are cheaper [87]. As a result, the share of agriculture in the gross domestic product has been rapidly decreasing despite a significant (but declining) population still being engaged in agriculture and availability of improved technologies for the agriculture sector [88].

Further, the interaction of the socio-economic sub-system with the prevention delay sub-system (as shown in CLDs) is yet to be appreciated by the health system, thus resulting in limited action on social determinants of NCDs. This continuous ignorance of socio-economic determinants of NCDs by the health system closely resonates with *Shifting the Burden* archetype (Fig 6E). This CLD model and its archetypes could provide critical insights to health system in addressing NCDs through comprehensive multi-sectoral action to address those socio-economic determinants of unhealthy diet and physical inactivity. Health sector are in a good position to advocate and accelerate actions on the social determinants of NCDs in relation to diet and physical activity and these CLDs and archetypes could be a starting point for action.

The research comprised of some key limitations. The broader scope of the study framework meant that we could only partially illustrate the key social determinants of unhealthy diet and physical inactivity risks in the context of Nepal. We have indicated within the paper that

localised qualitative and quantitative studies are needed to understand the dynamics interactions of the social determinants of NCDs and their key risks factors. Further, the CLDs can be improved as new evidence emerges, which is one of the key strength of systems thinking approach to understand and tackle a complex problem. A key methodological challenge was limited participation of local stakeholders during the sense-making workshops, which did affect the quality of sense-making workshop.

Conclusion

This paper has presented a combination of key social determinants influencing dietary practices and physical activity in Nepal. Health system actions were delayed in terms of regulating junk food sales and coordinating with other sector for improving physical activity. While diet and physical activity-related knowledge and practices as well as environmental factors were limited, the broader determinants such as socio-economic status accelerated the migration to urban areas and exposure of population (in particular, female and children) to an obesogenic environment. In particular, traditional dietary and physical activity practices were being displaced negatively affecting one's health. Decline in agricultural activities was affecting physical activity level, local food systems and ultimately economy. The health system could play a key role in raising awareness, monitoring the markets for unethical marketing of junk food, and coordinating with other sectors for strengthening local food systems, creating infrastructure that promotes physical activity and addressing socio-economic determinants of unhealthy diet and physical inactivity. Ministry of Health could advocate and show evidence of the need for such preventative action through the creation of a powerful national agency to facilitate inter-sectoral collaboration and achieve SDH focus.

Acknowledgement

The paper is derived from the PhD project of the first author and hence would like to acknowledge all the participants and supporters of the PhD study including Ministry of Health/Nepal, District Public Health Office Morang, District Public Health Office Bhaktapur. The authors would also like to thank research ethics committees of Massey University and Nepal Health Research Council. We would like to acknowledge Mr. Shiva Raj Mishra (PhD Student at University of Queensland) and Mr. Mohan Paudel (PhD Student at Flinders University) for their critical review and suggestions on this paper.

References

1. World Health Organization. Global Status Report on noncommunicable diseases 2014. World Health Organization, 2014.
2. Lachat C, Otchere S, Roberfroid D, Abdulai A, Seret FMA, Milesevic J, et al. Diet and Physical Activity for the Prevention of Noncommunicable Diseases in Low- and Middle-Income Countries: A Systematic Policy Review. PLOS Medicine. 2013;10(6):e1001465. doi: 10.1371/journal.pmed.1001465.
3. Lee IM, Shiroma EJ, Lobelo F, Puska P, Blair SN, Katzmarzyk PT. Impact of Physical Inactivity on the World's Major Non-Communicable Diseases. Lancet. 2012;380(9838):219-29. doi: 10.1016/S0140-6736(12)61031-9. PubMed PMID: PMC3645500.
4. Aryal KK, Mehata S, Neupane S, Vaidya A, Dhimal M, Dhakal P, et al. The Burden and Determinants of Non Communicable Diseases Risk Factors in Nepal: Findings from a Nationwide STEPS Survey. PloS one. 2015;10(8):e0134834. Epub 2015/08/06. doi: 10.1371/journal.pone.0134834. PubMed PMID: 26244512; PubMed Central PMCID: PMCPMC4526223.
5. Dhungana RR, Bista B, Pandey AR, de Courten M. Prevalence, clustering and sociodemographic distributions of non-communicable disease risk factors in Nepalese adolescents: secondary analysis of a nationwide school survey. BMJ open. 2019;9(5):e028263.
6. Kohl HW, 3rd, Craig CL, Lambert EV, Inoue S, Alkandari JR, Leetongin G, et al. The pandemic of physical inactivity: global action for public health. The Lancet. 380(9838):294-305. doi: 10.1016/S0140-6736(12)60898-8.
7. Graham H, White PCL. Social determinants and lifestyles: integrating environmental and public health perspectives. Public health. 2016;141:270-8. doi: https://doi.org/10.1016/j.puhe.2016.09.019.
8. World Health Organization. United Nations high-level meeting on noncommunicable disease prevention and control: NCD summit to shape the international agenda. New York, NY: Author; 2011 [cited 2013 May 15].
9. World Health Organization. Rio Political Declaration on Social Determinants of Health, Rio de Janeiro, Brazil. 2011. 2011.
10. World Health Organization. Sustainable Development Goals 2015 [cited 2015]. Available from: https://sustainabledevelopment.un.org/sdgs.
11. Solar O, Irwin A. A conceptual framework for action on the social determinants of health. Geneva: World Health Organization, 2010.
12. Marmot M, Bell R. Social determinants and non-communicable diseases: time for integrated action. Bmj. 2019;364:l251. doi: 10.1136/bmj.l251.
13. Donkin A, Goldblatt P, Allen J, Nathanson V, Marmot M. Global action on the social determinants of health. BMJ global health. 2017;3(Suppl 1):e000603-e. doi: 10.1136/bmjgh-2017-000603. PubMed PMID: 29379648.
14. Marmot M, Allen J, Goldblatt P, Boyce T, McNeish D, Grady M, et al. The Marmot review: Fair society, healthy lives. The Strategic Review of Health Inequalities in England Post-2010. 2010.
15. National Advisory Committee. The social, cultural and economic determinants of health in New Zealand: Action to improve health. Wellington: National Advisory Committee. 1998.
16. Marmot M, Allen J, Bell R, Bloomer E, Goldblatt P. WHO European review of social determinants of health and the health divide. The lancet. 2012;380(9846):1011-29.

17. Ball K, Carver A, Downing K, Jackson M, O'Rourke K. Addressing the social determinants of inequities in physical activity and sedentary behaviours. Health promotion international. 2015;30(suppl_2):ii8-ii19.
18. Fortune K, de Snyder NS, Galvão LAC, Murphy M. The Social Determinants of NCDs. Economic Dimensions of Noncommunicable Diseases in Latin America and the Caribbean. 2016:31.
19. Villalobos Dintrans P, Rodriguez L, Clingham-David J, Pizarro T. Implementing a Food Labeling and Marketing Law in Chile. Health Systems & Reform. 2020;6(1):e1753159. doi: 10.1080/23288604.2020.1753159.
20. Moodie R, Stuckler D, Monteiro C, Sheron N, Neal B, Thamarangsi T, et al. Profits and pandemics: prevention of harmful effects of tobacco, alcohol, and ultra-processed food and drink industries. The Lancet. 2013;381(9867):670-9.
21. Hallal PC, Andersen LB, Bull FC, Guthold R, Haskell W, Ekelund U. Global physical activity levels: surveillance progress, pitfalls, and prospects. The Lancet. 2012;380(9838):247-57. doi: 10.1016/S0140-6736(12)60646-1.
22. Singh DR, Sunuwar DR, Dahal B, Sah RK. The association of sleep problem, dietary habits and physical activity with weight status of adolescents in Nepal. BMC public health. 2021;21(1):938. doi: 10.1186/s12889-021-10985-5.
23. Paudel S, Owen AJ, Smith BJ. Exploration of Physical Activity Barriers and Facilitators Among Adults in Kathmandu, Nepal. Qualitative Health Research. 2021;31(6):1183-95. doi: 10.1177/1049732321993096.
24. Oli N, Vaidya A, Pahkala K, Eiben G, Krettek A. Knowledge, attitude and practice on diet and physical activity among mothers with young children in the Jhaukhel-Duwakot Health Demographic Surveillance Site, Nepal. PloS one. 2018;13(7):e0200329. doi: 10.1371/journal.pone.0200329.
25. Das P, Horton R. Physical activity—time to take it seriously and regularly. The Lancet. 388(10051):1254-5. doi: 10.1016/S0140-6736(16)31070-4.
26. Midgley G. Systemic Intervention for Public Health. American journal of public health. 2006;96(3):466-72. doi: 10.2105/AJPH.2005.067660. PubMed PMID: PMC1470519.
27. Sharma SR, Matheson A, Lambrick D, Faulkner J, Lounsbury DW, Vaidya A, et al. The role of tobacco and alcohol use in the interaction of social determinants of non-communicable diseases in Nepal: a systems perspective. BMC public health. 2020;20(1):1368. doi: 10.1186/s12889-020-09446-2.
28. Palinkas LA, Horwitz SM, Green CA, Wisdom JP, Duan N, Hoagwood K. Purposeful sampling for qualitative data collection and analysis in mixed method implementation research. Administration and Policy in Mental Health and Mental Health Services Research. 2015;42(5):533-44.
29. Smith J, Firth J. Qualitative data analysis: the framework approach. Nurse researcher. 2011;18(2):52-62.
30. Sterman JD. Business dynamics: Systems thinking and modeling for a complex world. Boston: Irwin/McGraw-Hill; 2000.
31. Microsoft. System requirements for Office. Available from: https://products.office.com/en-nz/office-system-requirements#coreui-contentrichblock-nnuz9v1.
32. Dedoose. Home 2016. Available from: http://www.dedoose.com/.
33. Ventana Systems Inc. Vensim Home USA: Ventana Systems, Inc.; 2015. Available from: http://vensim.com/.
34. Kim DH. Systems archetypes I: diagnosing systemic issues and designing high-leverage interventions: Pegasus Communications; 1993.

35. Department of Health Services. Annual Report 2015/16. Kathmandu, Nepal: Ministry of Health, 2016.
36. Department of Health and Family Welfare. Annual Report 2015/16. India: Ministry of Health and Family Welfare, 2016.
37. Medical Statistics Unit. Annual Health Bulletin. India: Ministry of Health, Nutrition and Indigenous Medicine, 2015.
38. Horton R. Offline: NCDs - why are we failing? The Lancet. 390(10092):346. doi: 10.1016/S0140-6736(17)31919-0.
39. Ministry of Finance. Budget Speech 2071/72 Nepal Kathmandu: Government of Nepal; 2015. Available from: http://mof.gov.np/uploads/cmsfiles/file/budget%20speech%202071-72_20140713125927.pdf.
40. Ministry of Health and Family Welfare. National action plan and monitoring framework for prevention and control of noncommunicable disease (NCDs) in India. Government of India; 2013.
41. MInistry of Health. NCD Action Plan Brazil: 2011-2022. Government of Brazil; 2011.
42. MInistry of Health. National Policy for the prevention and control of chronic non-communicable diseases in Ghana. Government of Ghana; 2012.
43. Ministry of Healthcare and Nutrition. The national policy and strategic framework for prevention and control of chronic non-communicable diseases. Government of Srilanka; 2009.
44. World Health Organization. Noncommunicable diseases progress monitor 2015. 2015.
45. Sharma SR, Page R, Matheson A, Lambrick D, Faulkner J, Mishra SR. Non-communicable disease prevention in Nepal: systemic challenges and future directions. Global health promotion. 2017:1757975917720800. doi: 10.1177/1757975917720800.
46. Reis RS, Salvo D, Ogilvie D, Lambert EV, Goenka S, Brownson RC, et al. Scaling up physical activity interventions worldwide: stepping up to larger and smarter approaches to get people moving. The lancet. 2016;388(10051):1337-48.
47. Sallis JF, Cerin E, Conway TL, Adams MA, Frank LD, Pratt M, et al. Physical activity in relation to urban environments in 14 cities worldwide: a cross-sectional study. The Lancet. 387(10034):2207-17. doi: https://doi.org/10.1016/S0140-6736(15)01284-2.
48. Ahmad B, Fouad FM, Elias M, Zaman S, Phillimore P, Maziak W. Health system challenges for the management of cardiovascular disease and diabetes: an empirical qualitative study from Syria. International journal of public health. 2015;60(1):55-62. doi: 10.1007/s00038-014-0594-2.
49. Willis-Shattuck M, Bidwell P, Thomas S, Wyness L, Blaauw D, Ditlopo P. Motivation and retention of health workers in developing countries: a systematic review. BMC health services research. 2008;8(1):247. doi: 10.1186/1472-6963-8-247.
50. Marten R, McIntyre D, Travassos C, Shishkin S, Longde W, Reddy S, et al. An assessment of progress towards universal health coverage in Brazil, Russia, India, China, and South Africa (BRICS). The Lancet. 2014;384(9960):2164-71. doi: https://doi.org/10.1016/S0140-6736(14)60075-1.
51. Balarajan Y, Selvaraj S, Subramanian SV. Health care and equity in India. The Lancet. 2011;377(9764):505-15. doi: https://doi.org/10.1016/S0140-6736(10)61894-6.
52. Alleyne G, Binagwaho A, Haines A, Jahan S, Nugent R, Rojhani A, et al. Embedding non-communicable diseases in the post-2015 development agenda. The Lancet. 2013;381(9866):566-74.
53. Bonita R, Magnusson R, Bovet P, Zhao D, Malta DC, Geneau R, et al. Country actions to meet UN commitments on non-communicable diseases: a stepwise approach. The Lancet. 2013;381(9866):575-84.

54. Beaglehole R, Ebrahim S, Reddy S, Voûte J, Leeder S. Prevention of chronic diseases: a call to action. The Lancet. 2008;370(9605):2152-7.
55. Misra A, Singhal N, Khurana L. Obesity, the metabolic syndrome, and type 2 diabetes in developing countries: role of dietary fats and oils. Journal of the American College of Nutrition. 2010;29(3 Suppl):289s-301s. Epub 2010/09/18. PubMed PMID: 20823489.
56. Pradeepa R, Anjana RM, Joshi SR, Bhansali A, Deepa M, Joshi PP, et al. Prevalence of generalized & abdominal obesity in urban & rural India- the ICMR - INDIAB Study (Phase-I) [ICMR - INDIAB-3]. The Indian journal of medical research. 2015;142(2):139-50. doi: 10.4103/0971-5916.164234. PubMed PMID: PMC4613435.
57. Vaidya A, Shakya S, Krettek A. Obesity Prevalence in Nepal: Public Health Challenges in a Low-Income Nation during an Alarming Worldwide Trend. International journal of environmental research and public health. 2010;7(6):2726-44. doi: 10.3390/ijerph7062726. PubMed PMID: PMC2905575.
58. Ramachandran A, Snehalatha C. Rising burden of obesity in Asia. Journal of obesity. 2010;2010.
59. Waqanivalu TK. Pacific islanders pay heavy price for abandoning traditional diet. Bulletin of the World Health Organization. 2010;88(7):484-5.
60. Kolahdooz F, Sheikholeslam R, Naghavi M, Abdollahi Z. Junk food consumption: an indicator of changing dietary habit in Iranian children. Asia Pacific journal of clinical nutrition. 2004;13.
61. Hassan Wassef H. Food habits of the Egyptians: newly emerging trends. 2004.
62. Jacobs A, Richtel M. How big business got Brazil hooked on junk food. New York Times, Sept. 2017;16:2017.
63. Stuckler D, Nestle M. Big Food, Food Systems, and Global Health. PLOS Medicine. 2012;9(6):e1001242. doi: 10.1371/journal.pmed.1001242.
64. Sadeghirad B, Duhaney T, Motaghipisheh S, Campbell NRC, Johnston BC. Influence of unhealthy food and beverage marketing on children's dietary intake and preference: a systematic review and meta- analysis of randomized trials. Obesity Reviews. 2016;17(10):945-59. doi: doi:10.1111/obr.12445.
65. RECPHEC. Rapid assessment on media coverage of junk food and its content analysis on selected Nepali and Indian Television Channels. Kathmandu, Nepal: Resource Centre for Primary Health Care (RECPHEC), 2013.
66. Dixon HG, Scully ML, Wakefield MA, White VM, Crawford DA. The effects of television advertisements for junk food versus nutritious food on children's food attitudes and preferences. Social Science & Medicine. 2007;65(7):1311-23. doi: https://doi.org/10.1016/j.socscimed.2007.05.011.
67. Hastings G, McDermott L, Angus K, Stead M, Thomson S. The extent, nature and effects of food promotion to children: a review of the evidence. Geneva: World Health Organization. 2006;20.
68. Nestle M. Food politics: How the food industry influences nutrition and health: Univ of California Press; 2013.
69. Jolly R. Marketing Obesity?: Junk Food, Advertising and Kids: Parliamentary Library; 2011.
70. Magnusson RS. Obesity prevention and personal responsibility: the case of front-of-pack food labelling in Australia. BMC public health. 2010;10(1):662. doi: 10.1186/1471-2458-10-662.
71. Tripathy JP, Thakur JS, Jeet G, Chawla S, Jain S, Prasad R. Urban rural differences in diet, physical activity and obesity in India: are we witnessing the great Indian

equalisation? Results from a cross-sectional STEPS survey. BMC public health. 2016;16(1):816. doi: 10.1186/s12889-016-3489-8.
72. Anjana RM, Pradeepa R, Das AK, Deepa M, Bhansali A, Joshi SR, et al. Physical activity and inactivity patterns in India – results from the ICMR-INDIAB study (Phase-1) [ICMR-INDIAB-5]. The international journal of behavioral nutrition and physical activity. 2014;11:26-. doi: 10.1186/1479-5868-11-26. PubMed PMID: PMC3974063.
73. Moniruzzaman M, Zaman MM, Islalm M, Ahasan H, Kabir H, Yasmin R. Physical activity levels in Bangladeshi adults: results from STEPS survey 2010. Public health. 2016;137:131-8.
74. Ng N, Hakimi M, Van Minh H, Juvekar S, Razzaque A, Ashraf A, et al. Prevalence of physical inactivity in nine rural INDEPTH Health and Demographic Surveillance Systems in five Asian countries. Global health action. 2009;2(1):1985.
75. Kimokoti RW, Fung TT, Millen BE. Chapter 7 - Dietary Patterns/Diet and Health of Adults in Economically Developing Countries. Bioactive Food as Dietary Interventions for the Aging Population. San Diego: Academic Press; 2013. p. 83-107.
76. Pou SA, del Pilar Díaz M, De La Quintana AG, Forte CA, Aballay LR. Identification of dietary patterns in urban population of Argentina: study on diet-obesity relation in population-based prevalence study. Nutrition Research and Practice. 2016;10(6):616-22. doi: 10.4162/nrp.2016.10.6.616. PubMed PMID: PMC5126411.
77. Popkin BM, Adair LS, Ng SW. NOW AND THEN: The Global Nutrition Transition: The Pandemic of Obesity in Developing Countries. Nutrition reviews. 2012;70(1):3-21. doi: 10.1111/j.1753-4887.2011.00456.x. PubMed PMID: PMC3257829.
78. Prochaska JO, Velicer WF. The transtheoretical model of health behavior change. American journal of health promotion. 1997;12(1):38-48.
79. Scharff RL. Obesity and hyperbolic discounting: Evidence and implications. Journal of Consumer Policy. 2009;32(1):3-21.
80. Lawton J, Ahmad N, Hanna L, Douglas M, Hallowell N. 'I can't do any serious exercise': barriers to physical activity amongst people of Pakistani and Indian origin with Type 2 diabetes. Health education research. 2006;21(1):43-54. doi: 10.1093/her/cyh042.
81. Harvard Health Publishing. An Introduction to Yoga. Harvard Medical School, 2016.
82. Hallal PC, Matsudo SM, Matsudo VKR, Araújo TL, Andrade DR, Bertoldi AD. Physical activity in adults from two Brazilian areas: similarities and differences. Cadernos de saude publica. 2005;21:573-80.
83. Knuth AG, Bacchieri G, Victora CG, Hallal PC. Changes in physical activity among Brazilian adults over a 5-year period. J Epidemiol Community Health. 2010;64(7):591-5. Epub 2009/08/27. doi: 10.1136/jech.2009.088526. PubMed PMID: 19706621.
84. Singh A, Purohit B. Physical activity, sedentary lifestyle, and obesity among Indian dental professionals. Journal of Physical Activity and Health. 2012;9(4):563-70.
85. Keeley B, Wright L, Condit CM. Functions of health fatalism: fatalistic talk as face saving, uncertainty management, stress relief and sense making. Sociology of health & illness. 2009;31(5):734-47.
86. Balarajan Y, Villamor E. Nationally representative surveys show recent increases in the prevalence of overweight and obesity among women of reproductive age in Bangladesh, Nepal, and India. The Journal of nutrition. 2009;139(11):2139-44. Epub 2009/09/25. doi: 10.3945/jn.109.112029. PubMed PMID: 19776182.
87. Bishwajit G. Nutrition transition in South Asia: the emergence of non-communicable chronic diseases. F1000Research. 2015;4:8. doi: 10.12688/f1000research.5732.2. PubMed PMID: PMC4706051.

88. World Bank. Data: Agriculture, forestry, and fishing, value added (% of GDP) 2018. Available from: https://data.worldbank.org/indicator/NV.AGR.TOTL.ZS.

Supporting information

S1 File. COREC Checklist

S2 File. Interview and FGD guidelines

Fig1. Systemic Intervention design of the study

Click here to access/download;Figure;Fig1. Systemic Intervention design of the study.tif

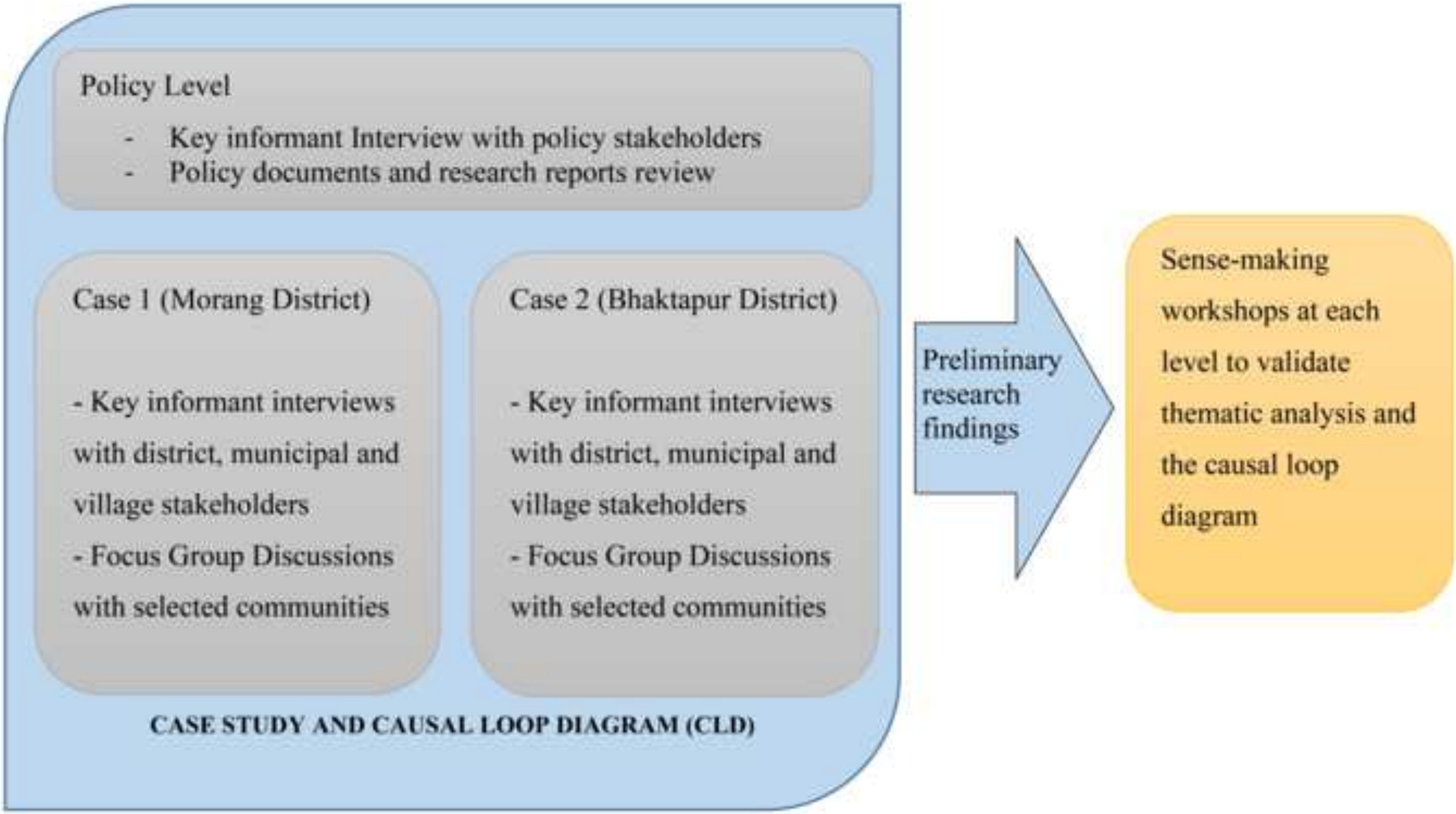

Fig2. Study framework adapted from the SDH Framework

Click here to access/download;Figure;Fig2. Study framework adapted from the SDH Framework.tif

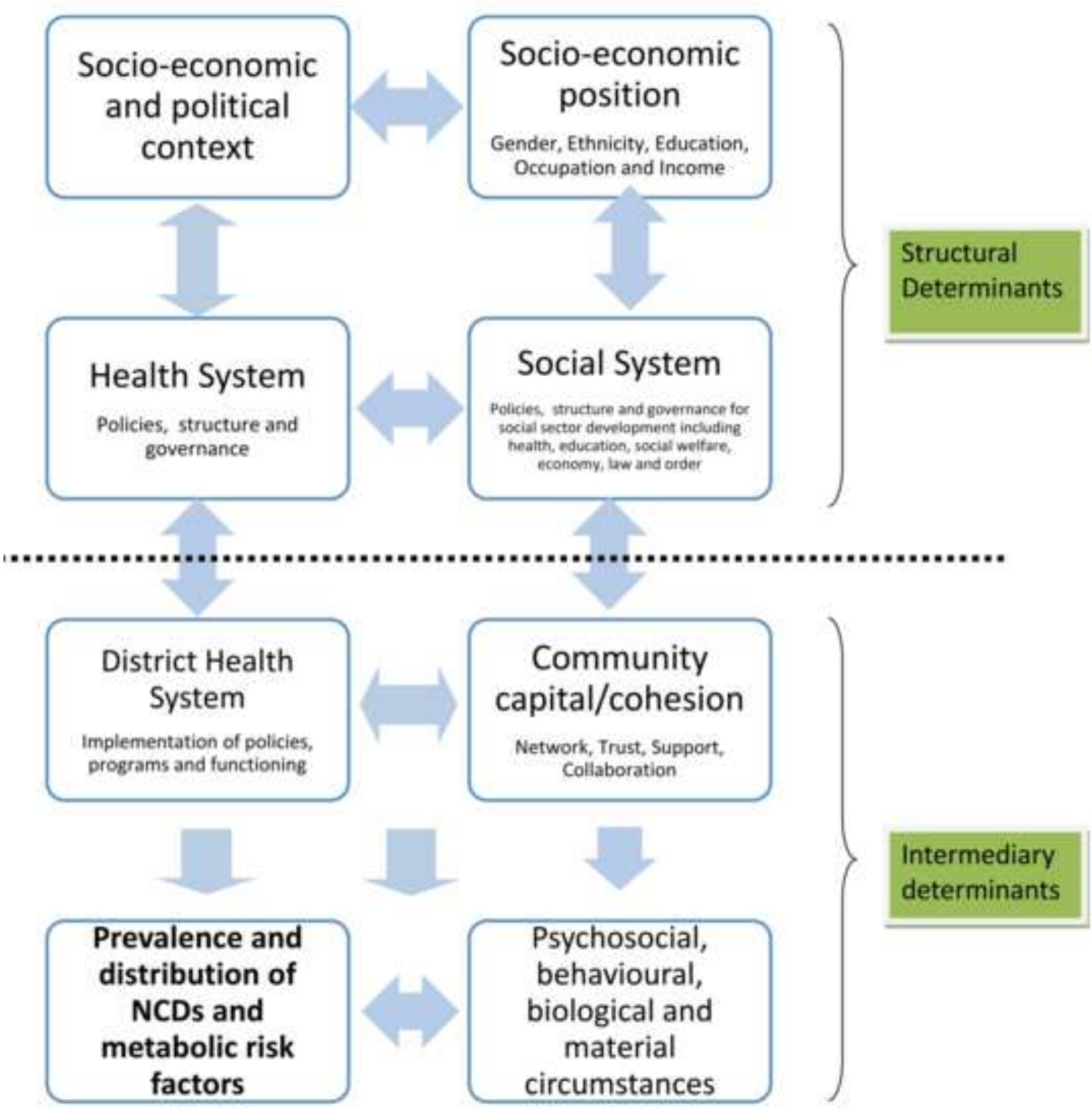

Fig3. Prevention delay subsystem Click here to access/download;Figure;Fig3. Prevention delay subsystem.tif

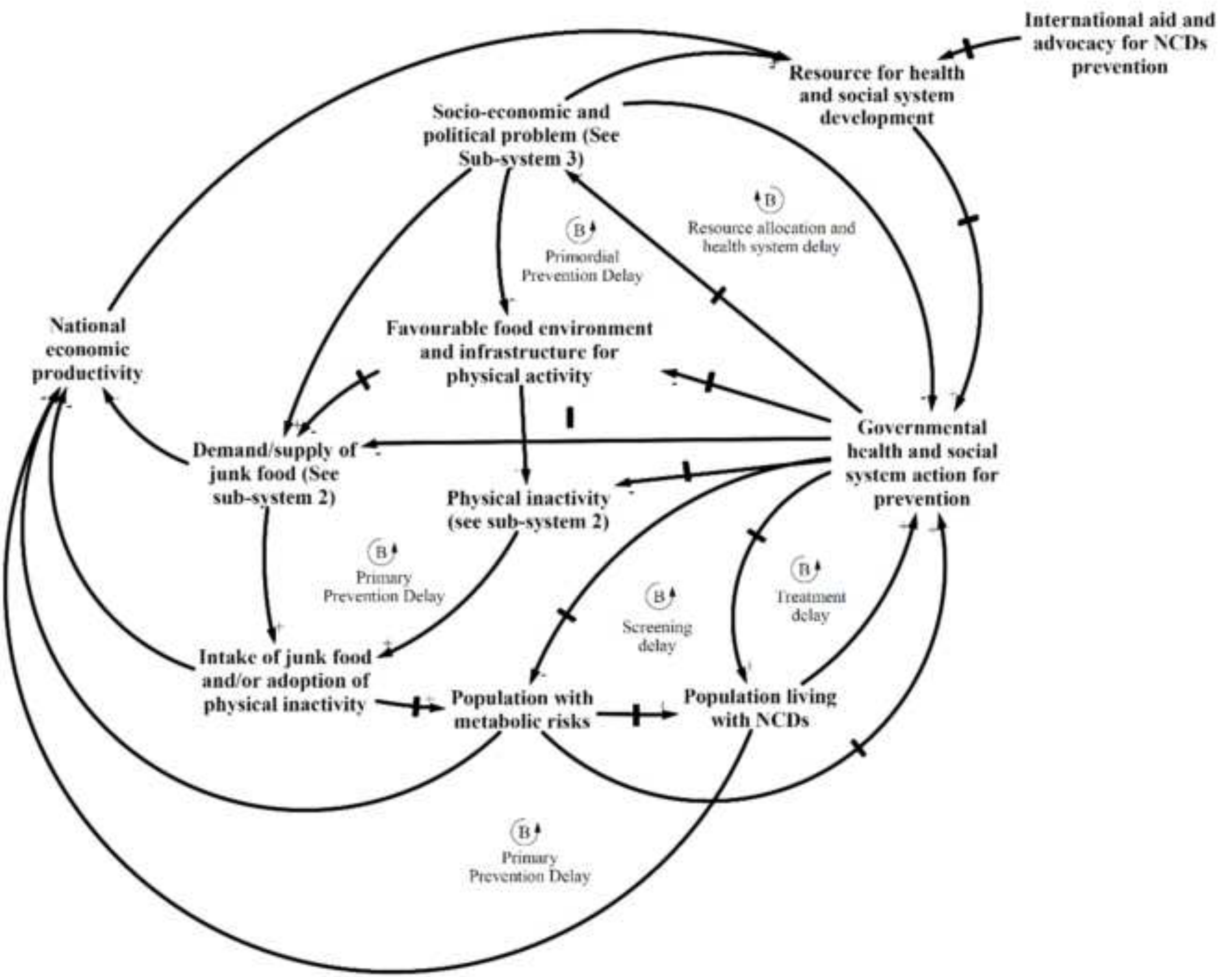

Fig4. Demand supply subsystem

Click here to access/download;Figure;Fig4. Demand supply subsystem.tif

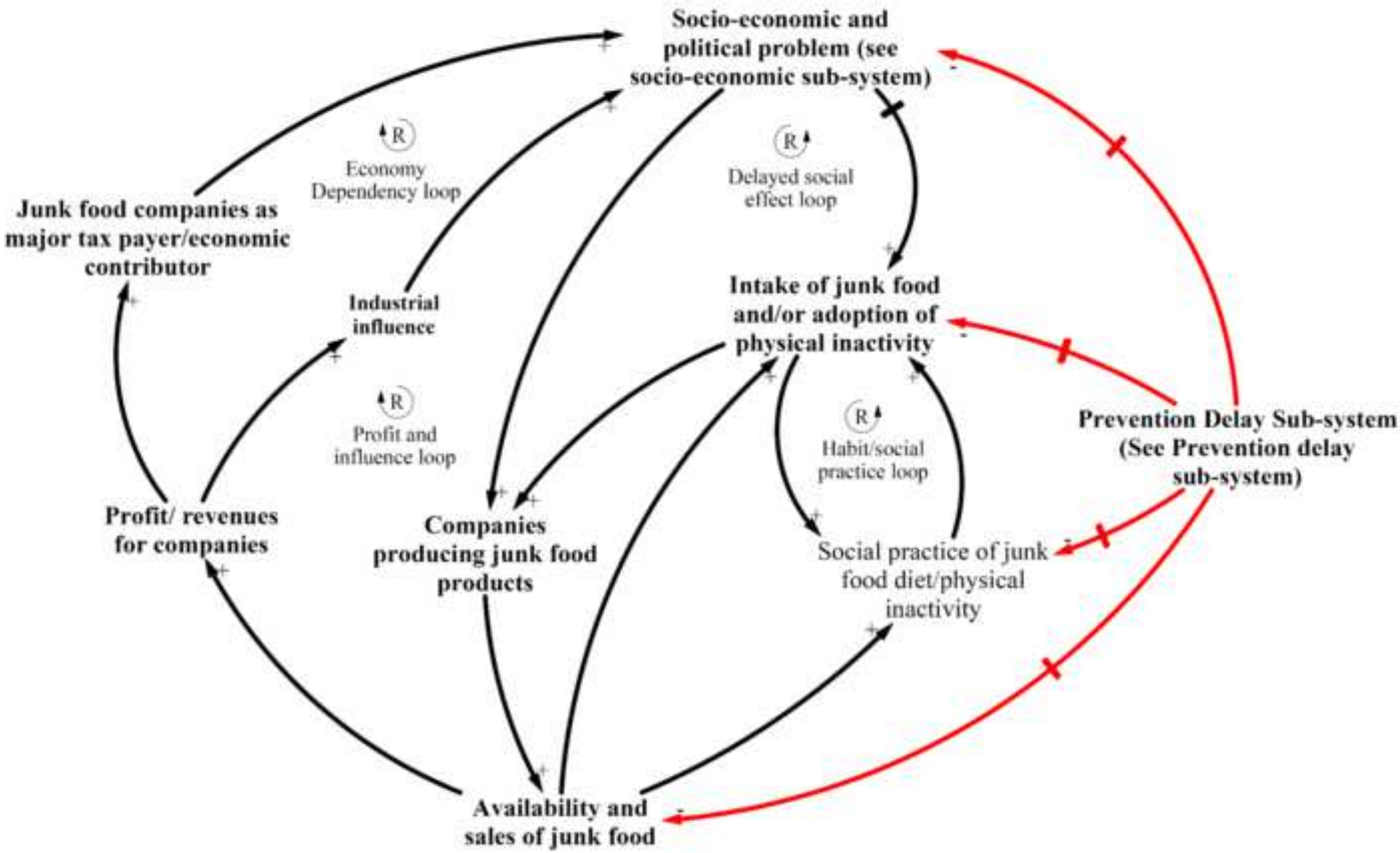

Fig5. Socioeconomic influence subsystem

Click here to access/download;Figure;Fig5. Socioeconomic influence subsystem.tif

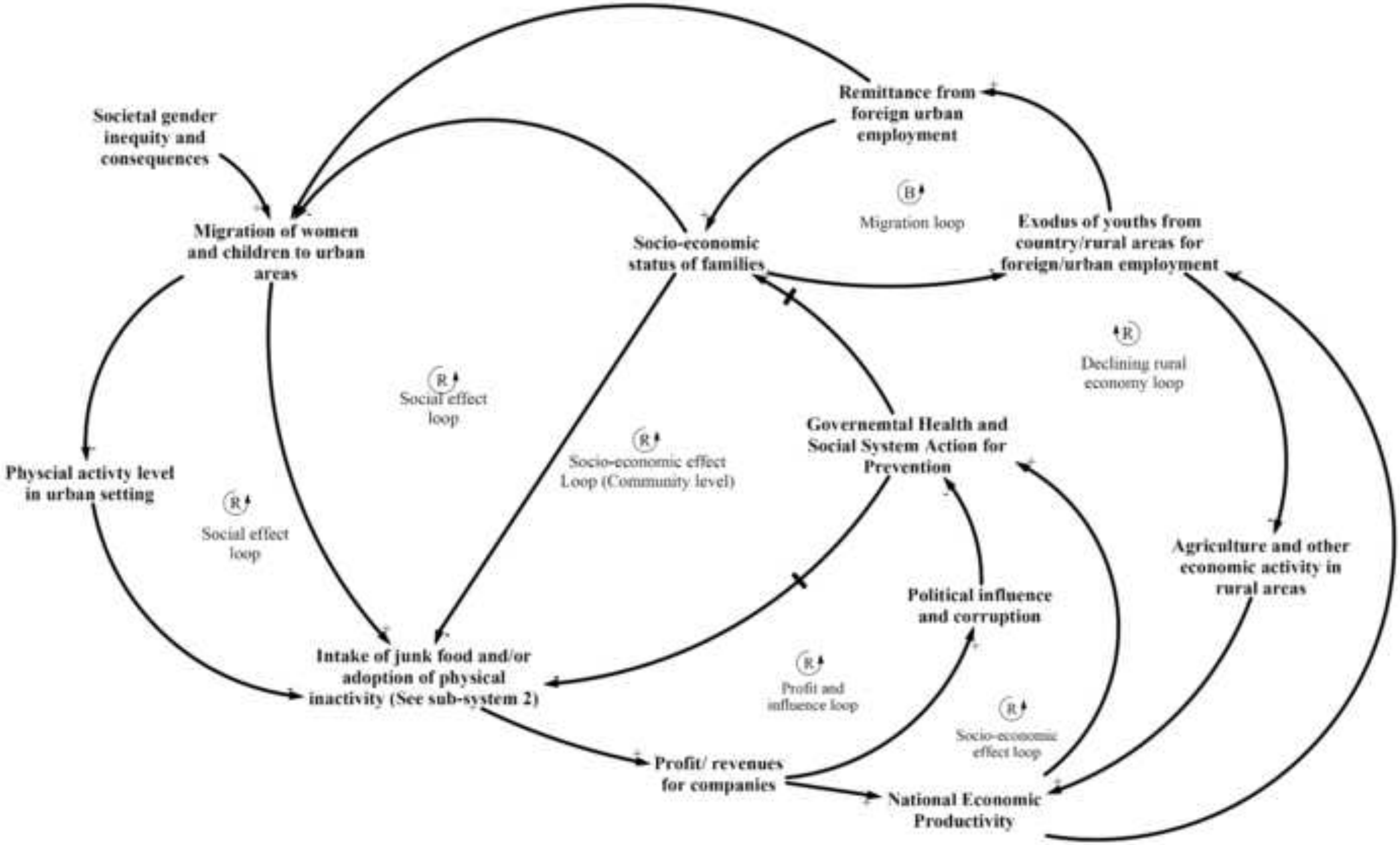

Fig6. System archetypes Click here to access/download;Figure;Fig6. System archetypes.tif

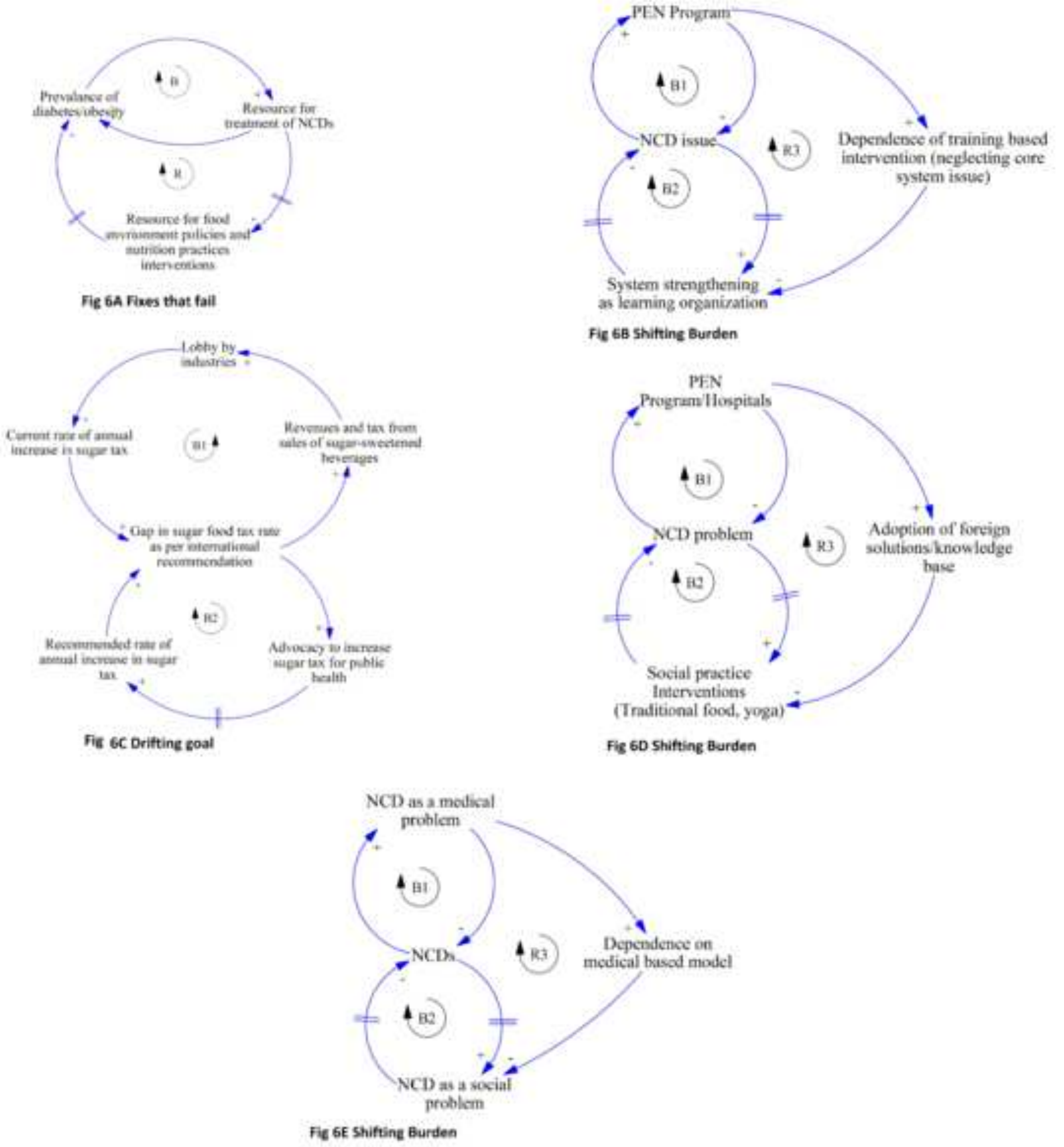

S2 File. Interview and FGD guidelines

Click here to access/download
Supporting Information
S2 File Interview and FGD guidelines.docx

S1 File. COREC Checklist

Click here to access/download
Supporting Information
COREC Checklist Paper 3.rtf

~~Title:~~ Dietary practices, physical activity and social determinants of non-communicable diseases in Nepal: a systemic analysis

Formatted: Font: 18 pt

~~**Short title:** Dietary practices, physical activity and social determinants of NCDs~~

~~**Authors name (email):**~~

Sudesh ~~Raj~~ Sharma[1,2]* ~~(yoursudesh@gmail.com),~~

Anna Matheson[3] ~~(anna.matheson@vuw.ac.nz),~~

Danielle Lambrick[4] ~~(D.M.Lambrick@soton.ac.uk),~~

James Faulkner[5] ~~(),~~

David W. Lounsbury[6] ~~(David.Lounsbury@einstein.yu.edu)~~

~~,~~ Abhinav Vaidya[7] ~~(dr.abhinavaidya@gmail.com),~~

Rachel Page[2] ~~(r.a.page@massey.ac.nz)~~

Formatted: Tab stops: Not at 2.1"

Formatted: Tab stops: Not at 1.67"

Formatted: Tab stops: 1.67", Left

Formatted: Tab stops: 1.67", Left + Not at 2.1"

~~**Authors affiliations**~~

[1] DIYASU Community Development Centre, Biratnagar, Morang, Nepal

[2] Massey University, Wellington, Wellington Region, New Zealand

[3] Victoria University of Wellington, Wellington, Wellington Region, New Zealand

[4] University of Southampton, Southampton, Hampshire, UK

[5] University of Winchester, Winchester, Hampshire, UK

[6] Albert Einstein College of Medicine, Bronx, New York, US

[7] Kathmandu Medical College, Kathmandu, Bagmati, Nepal

*Corresponding author

~~Sudesh Raj Sharma~~

Email: yoursudesh@gmail.com

(SS)

Formatted: Font: Not Bold

~~**Authors contributions**~~

~~SRS conceived and drafted the initial manuscript. AM, JF, DL, DWL, AV and RP all critically reviewed and revised the initial manuscript. SRS prepared the final manuscript. All authors read and approved the final manuscript.~~

b

b

Abstract

Unhealthy dietary habit and physical inactivity are major risk factors of non-communicable diseases (NCDs) globally. ~~**Background:**~~ The objective of this paper was to describe the role of dietary practices and physical activity ~~and~~in the ~~in the~~ interaction ~~witho~~of~~of~~ the social determinants of ~~non-communicable diseases (~~NCDs~~)~~ in Nepal, a developing economy.

~~**Method:**~~ The study was a qualitative study design- involving two districts in Nepal, ~~guided by systemic intervention methodology. Qualitative data were collected through~~ and collection ~~of data~~whereby data was collected via key informant interviews (n=63) and focus group discussions (n= 12). Thematic analysis of the qualitative data was performed ~~carried out~~ and a causal loop diagram was built to~~,~~ ~~and case study-based thematic analysis was carried out. Causal loop diagram was utilised to~~ illustrate the dynamic interactions of the social determinants based on the themes. The- study also involved sense-making sessions with policy level and local stakeholders.

~~**Findings:**~~ Four key interacting themes emerged from the study: i)~~.~~ d~~D~~ietary and physical activity practices have shifted significantly at community level contributing to increased risk of NCDs; ii)~~.~~ t~~T~~he practice of healthy and locally produced meals was gradually being displaced by junk food and linked to junk food availability and declining agricultural activities; iii)~~.~~ h~~H~~ealth system was ineffective in preventing unhealthy practices; and, iv) g~~.~~ ~~G~~ender and socio~~al-~~-economic factors were driving the ~~poor~~unhealthy dietary practices and physical inactivity.

~~**Conclusion:**~~ While the current dietary and physical activity-related practices within communities were poor, the broader determinants such as socio-economic circumstances and gender further fuelled such practices. The health system has potential to play an effective role in the prevention of the behavioural and social determinants of NCDs.

Formatted: Normal

Formatted: Space After: 0 pt

Background

~~Poor~~Unhealthy dietary habit and physical inactivity have been identified as key risk factors in increasing non-communicable diseases (NCDs) in developing countries [1, 2]. It has been estimated that dietary and physical activity risks attribute to approximately 5.3 million premature deaths annually [3]. In Nepal, the last Stepwise approach to surveillance (STEPS) survey has shown that about 99% of people did not consume sufficient fruits and vegetables but only 3% did not have adequate physical activity [4]. However, the problem of physical inactivity was more pervasive in the urban and peri-urban areas with more than 5% of urban respondents categorised as physically inactive [4]. A recent study showed that inadequate physical activity among adolescents was 85%, indicating the shift towards physical inactivity in Nepal [5].

Increasingly, there is evidence relating to socio-economic and environmental determinants influencing diet and physical activity, often collectively termed social determinants [6, 7]. Global health agencies and experts have been pushing the agenda of NCDs prevention in both developed and developing nations through focusing on the social determinants of health [8-12]. In 2008, the World Health Organization proposed the landmark *Social Determinants of Health Framework,* which highlighted the association of proximal and distal determinants~~complex causal mechanism~~ of ~~public~~ health~~ issues~~ and suggested directions for actions [11]. Since then, there has been increasing focus in understanding and taking action on the social determinants of complex health issues such as NCDs globally [13]. However, developing countries are far behind in their understanding and actions on the social determinants of health compared to developed countries [13-17]. On a positive note, some Latin American and Caribbean countries ~~are taking~~have initiated significant steps in addressing the social determinants of NCDs [18, 19].

Global evidence has suggested that physical inactivity as well as junk food consumption is rapidly increasing due to social, environmental and economic influences [6, 7, 20]. Rapid urbanisation and changing nature of work (sedentary nature and increasing mechanisation of physical work) are impacting physical activity and dietary patterns [6, 21]. A Lancet review has suggested that physical inactivity is increasing among lower socio-economic groups [21]. Moodie et. al. suggested that transnational companies are driving the junk food epidemic globally, resulting in increasing NCDs, even in developing countries [20]. ~~A study~~Studies ~~in~~

from Nepal have~~s~~ shown that urban population have an increased risk of chronic diseases due to ready access to junk food and limited avenues for physical activity [4, 22-24]. Further, limited policy and multi-sectoral action on healthy diet and optimal physical activity promotion have resulted in increase in NCDs and their risks [2, 25]. However, there is limited evidence or analysis of the links between social determinants and NCDs mediated by diet and physical activity in Nepalese and developing countries context. This paper assesses the role of dietary practices and physical activity in the interaction of the social determinants of NCDs in Nepal.

Methods

The study design was qualitative study based on systemic intervention (SI) methodology (Fig~~figure~~ 1) [26, 27]. SI adopts the critical systems thinking approach and stresses on using multiple methods to understand the complex problems. A combination of case study and system dynamics (causal loop diagram) methods, along with participatory sense-making workshops, were adopted within the research design.

Fig 1. Systemic intervention study design to study social determinants of NCDs in Nepal (Image republished from authors' open access article [27])

Study area and study participants

The study was conducted in Nepal from July until October 2016. Two districts (Bhaktapur and Morang) from Nepal were purposively selected as case districts. Bhaktapur is a hilly district near the capital city of Nepal and has a sub-tropical cold and humid climate. Morang is a plain district located in the eastern part of Nepal and has a sub-tropical hot and humid climate. Within each case district, one municipality and two village development committees (VDC) were selected for community level data collection.

Community members affected by NCDs and key stakeholders from community, district and policy level were the participants of the study. The key stakeholders were purposively selected based on their current engagement (or potential engagement) in NCDs prevention and health promotion in the case districts and at policy level in Nepal. They included members of *NCDs Multi-sectoral Action Plan Steering Committee*, academia, non-government organisations, District Health Officer, Local Development Officer, community-

based organisations, local health workers and village leaders. The communities for focus group discussion (FGD) were identified through the referral of respective district health offices and local health institutions.

Data collection

Data collection involved semi-structured interviews with key stakeholders from the case districts and policy level. Policy level data were collected to supplement the information from case districts. ~~We developed the~~S ~~s~~emi-structured interview guideline so developed were~~,~~ ~~which was~~ guided by the adapted social determinants of health framework (Fig~~figure~~ 2).

Fig 2. Adapted Social Determinants of Health Framework for the study of the social determinants of NCDs in Nepal (Image republished from authors' open access article [27])

The sampling strategy for the study entailed identifying and participating individuals knowledgeable and experienced in the issue of interest i.e. NCDs prevention at case district and policy level in Nepal. The purpose of the purposive sampling was to capture the different perspectives and contexts on the NCDs prevention issue guided by the study framework (F~~f~~ig~~igure~~ 2). Purposive sampling is often used in qualitative studies for identifying and interviewing potential information-rich subjects on a complex issue like NCDs [28].~~We adopted the~~ ~~"m~~M*aximum variation*~~"~~ strategy was adopted to ensure diverse perspectives across the health and social sectors in Nepal were captured relating to complex issue of NCDs ~~to investigate any complex issue that emerged from across the sectors~~ [28]. Thirty-nine participants were interviewed from the two case study sites (Bhaktapur and Morang) and 24 participants from policy level. All of the interviews were carried out by the first author in Nepali language. The time of interview ranged from 30 minutes to one hour.

Further, 12 focused group discussions (FGDs) were conducted within the selected municipalities and VDCs of the case districts. Each FGD included five to 10 community people experiencing and/or caring for family members with NCDs and their metabolic risks. The purpose of FGDs was to capture negotiated views on NCDs and risks as experienced by individuals, families and community members belonging to different socio-economic groups. Therefore, in each VDC/municipality, two FGDs were conducted with one group representing a socio-economically disadvantaged community and the other a more

Formatted: Font: Italic

Formatted: Font: Italic

advantaged/mixed community. The FGDs were facilitated by the first author with the help of local volunteers. The duration of FGDs ranged from 45 minutes to one hour.

Data management and analysis

~~We first transcribed~~ T~~t~~he interviews and FGDs audio recordings were first transcribed in Nepali and then translated them into English for coding and thematic analysis. ~~We utilised the~~A *Framework Approach* was used to undertake~~to carry out~~ the thematic analysis guided by the study framework (Fig~~figure~~ 2) [29]. Themes were identified, grouped and coded by using both a deductive approach based on the study framework as well as more inductive approaches to allow for emergent themes. Case analysis helped in framing the qualitative and quantitative data for the local context and iterating on ~~causal linkages~~association of various social determinants. ~~We then developed a system map~~A systems map or c~~C~~ausal Loop Diagram (CLD) was developed to illustrate the ~~causal linkage and mechanism~~association of the social determinants of NCDs indicated by thematic analysis. Causal loop diagramming is a system dynamics technique to build a system map of a problem of interest that illustrates the causal ~~mechanism~~ association through feedback structures that are loopy in nature [30]. CLD consists of two types of feedback loops: balancing and reinforcing loops, and these loops have variables connected by uni-directional arrows with either positive or negative signs on them. A positive sign indicates that if the independent variable increases or decreases, the dependent variable also increases or decreases in the same direction. A negative sign indicates the exact opposite meaning i.e. if independent variable increases, then the dependent variable decreases and vice versa. The balancing loop is a goal-seeking loop, which is indicated by *B* within CLD and indicates the stabilising feature of the loop. In general, causal loops with an intervention are of a balancing nature that tried to mitigate the problem. On the other hand, reinforcing loops (indicated by *R* within CLD) depict a mechanism that produces a result that influences more of the same action, thus resulting in either growth or decline. An example of reinforcing loop can be the vicious cycle of poverty and ill health. *Dedoose (Socio-cultural Research Consultants)* and *MS Excel 2016 (Microsoft)* [31] were used to manage the qualitative data [32] and *Vensim (Ventana Systems Inc.)* was used to build CLD [33].

Stakeholder Validation

Stakeholder validation was conducted through organising three sense-making workshops, two at respective case districts and one at national level during January/February, 2018. The workshops helped to further enrich the analysis through the feedback and suggestions from the stakeholders. These workshops, indeed, became opportunities to sensitise the stakeholders about the impending epidemic in Nepal. The workshops proved useful in reaching the purpose of this research and the methodological stance of systemic intervention methodology.

Ethical Consideration

Formal ethical approvals were taken from the Massey University Human Ethics Committee (SOA 16/37) and Nepal Health Research Council Ethics Committee (Reg. no. 163/2016) respectively. All participants, and in particular disadvantaged and less literate groups, were clearly informed about the purpose of the study. All participants were provided with an information sheet, and a voluntary written consent was obtained from all participants of semi-structured interviews and focus group discussions.

Findings

Four key interacting thematic areas relating to dietary practices and physical activity emerged, which were utilised to develop a causal loop diagram and demonstrate dynamic complexity.

Theme 1: Practice relating to diet and physical activity contributing to increased risk

Key informants and focus group participants indicated that current food habits among most Nepalese were not balanced, which was potentially leading to the problem of NCDs. A focus group participant from rural Morang shared:

> *"Nepalese food is taken three times a day. And there is rice every time, even in the snack. That is why now, 2 out of 4 people of a family have high sugar level." (ID: 69)*

Some participants expressed pre-existing and predominant beliefs, which was sustaining such ~~poor~~unhealthy dietary practices.

> *"We eat enough food (at night). Since that has to withstand us up to 10 am in the morning." (ID: 69; Rural Morang; FGD Participant)*

One key observation highlighted by participants from the case districts was that the traditional practice of healthy eating was being gradually displaced by westernised practice of eating, which is unhealthy for the Nepalese context. A FGD participant from rural Bhaktapur lamented:

> *“We used to have “Dhido” in the morning and in the evening. We used to run home from school during lunchtime, have the lunch and return back to school. But these days vehicles come to pick up and drop our children and there is no time to prepare lunch for the children. So junk foods are easy.” (ID: 76)*

A policy level key informant explained that overwhelming availability and appeal of junk foods were affecting local dietary practices.

> *“In case of food habit, our raw food/fruits habits have been changed to junk food based diet. Junk food are readily available, easy to cook and do not need much effort. There is an increase in the use of junk food from a very early age.” (ID: 3; Policy Stakeholder; Health)*

Some participants stressed that the Nepalese culture and traditions have potential to promote physical activity and healthy diet but they continue to be ignored. Offering prayers to different temples in a single walk (Newari culture), yoga and meditation (Hindu culture), *Namaz* prayer (ritual Muslim prayer, which also involves different body movements) were stressed by participants as practices that have physical activity significance.

> *“In context of our geographically and culturally diverse country, we have our own traditions of practicing yoga, meditations, bipasyana etc. but these aren’t practiced in our daily schedule.” (ID: 12; Policy Stakeholder; Health)*

Participants also reported the decline in physical activities like walking due to increasing use of motor vehicles in the communities.

> *“People who used to walk now use bicycle and motorbike now and that makes easy for them. Because of that too, it has been decreased now.” (ID: 66; Urban Morang; FGD Participant)*

A case study district stakeholder elaborated that people often resort to physical activity practice, including yoga and morning walks, after they start suffering from diseases or metabolic risk.

"If we ask 100 people who come to Yoga if they do not have any such disease or conditions, only few hands would raise up." (ID: 30; Bhaktapur District Stakeholder; Non-health)

Theme 2: Health sector issues delaying actions to improve dietary practice and physical activity

Key policy stakeholders shared that health sector has historically considered NCDs as a curative care agenda instead of a multi-sectoral prevention agenda. This has led to current lack of concrete structure and leadership relating to NCDs prevention which is creating a delay in coordinated action for nutrition and physical activity promotion.

"Our health system is resisting to adapt to addressing NCDs issues from health promotion and prevention aspect." (ID: 18; Policy Stakeholder; Health)

As a result, district stakeholders reported that the district health system and below have limited NCDs prevention-related activities in addition to systemic health system issues. Health workers lacked training in NCDs prevention and the office had limited resources at their disposal. A district stakeholder shared how the current health system was more focused on curative care than preventative action.

"Our [Primary Health Care] network is very good. But, it is less focus on primary prevention compared to curative. Our system can do more primary prevention." (ID; 25; Bhaktapur District Stakeholder; Health)

A key policy stakeholders expressed limited health sector intervention in curbing marketing and promotion of junk food leading to current junk food based dietary practice and displacing the traditional diet.

"Second one is food habit where major issue is aggressive advertisement of junk foods. What is happening is, gradually we are leaving our indigenous food habits and shifting towards the trend of consumption of junk foods may be as a fashion or may be as a status symbol." (ID: 9; Policy Stakeholder; Non-health)

A rural health worker shared how the dietary practice has shifted in the last 10 years as follows:

"Nowadays people are eating lot of fast food. Ten years earlier people used to have home beaten rice and beans; corn (pop-corn) and would have a balance diet. But now

people do not eat at home; they eat outside and mostly junk food." (ID: 35; Rural Bhaktapur; Health Worker)

In addition, participants stressed that increased motorisation and lack of systemic infrastructure were leading to limited physical activity in the case studies. There has been a rapid development in road infrastructure and motorisation of public and private transport systems. However, due to lack of awareness regarding active travel and luxury-seeking behaviour, this has been contributing to physical inactiveness to some extent.

> *"These days, vehicles are available right in our doorsteps. Now instead of walking to the place that takes 30 minutes, we wait an hour for the vehicles to take us there." (ID: 65; Urban Morang; FGD Participants)*

Urban areas of Nepal have poor urban planning, leading to limited green and open spaces for promoting physical activity among the residents. Though there are policies and regulations relating to urban and housing design in place, residents simply ignore them and the regulations are not strongly implemented enough to have any meaningful impact. This is leading to cumulative effect of limited space as explained by a policy stakeholder:

> *"What has happened is lack of open space has become an increasing problem because of population growth and exposure to open space is diminishing /decreasing. This leads to decrease in people's habit of roaming, walking, exercise and lack of good/high oxygen concentration. This trend is slowly fading away even in rural areas and condition has worsened in urban areas." (ID: 11; Policy Stakeholder; Health)*

Some participants linked limited physical activity with system ignorance and limited promotion of yoga and traditional physical activity. Often the system was not focusing on instilling habits of physical activities among the young people.

> *"We ourselves are responsible for the current state of yoga. We couldn't direct the young generations to a correct track." (ID: 52; District Stakeholder Morang; Non-health)*

Theme 3: Gendered-related norms and cultural shifts affecting physical activity and dietary practice

There were some revelations about the influence of gender on the physical activity and dietary practice. Participants shared that in urban areas, females tended to be physically inactive and overweight/obese.

> *"In urban and semi urban areas obesity is seen more in married female." (ID: 5; Policy level)*

However, there were some positive actions being initiated within urban communities where more females compared to male were engaged in yoga.

> *"You may see that many females come to the yoga and are often married. Not many males come; probably due to complacency and laziness." (ID: 44; Urban Bhaktapur; Social Worker)*

Similarly, participants shared that more and more families of foreign employed males were migrating to urban centres for a better life. As a result, female members within such families were detached from their regular agricultural works in the rural context and confined to limited space rented houses, which significantly reduced their physical activity.

> *"If we talk about those families whose members are working abroad, their families now have started living in the market areas and depend upon market for every necessities including the vegetables which once they themselves used to grow." (ID: 7; Policy Stakeholder; Health)*

In rural areas of case districts, women who used to be engaged in agricultural activities and animal rearing have rapidly given up such practices.

> *"In our time we used to run to the field soon after having the meal. We used to cultivate different crops and plants like maize, mustard and many more. We used to grow mustard here like in Chitwan but now there are only bushes and grasses in those lands." (ID: 76; Rural Bhaktapur; Female FGD Participant)*

Theme 4: Migration affecting agriculture sector and exposing vulnerable families to obesogenic environment

The participants pointed that migratory movement of youths and adults and their families from rural to urban and foreign countries was one of the key reason for rapid decline of agriculture activities. The migration created a shortage of agricultural workforce in rural areas. The migration was mainly driven by limited economic opportunities in rural areas and

prospect of better opportunities in urban and foreign land. A policy stakeholder noted that foreign migration was having double whammy effects in terms of affecting the agriculture sector as well as exposing vulnerable families to unhealthy lifestyle.

"At least one member of a household is earning in foreign land. Now the proportion of population involved in agriculture has decreased in recent times. People's profession has changed. Along with the change in profession, people's lifestyle has also changed." (Policy Stakeholder; ID: 6; Health)

A rural stakeholder further added that the decline in agricultural activities was directly resulting in increased dependency on commercially available food and decrease in physical activity in rural areas.

"Importantly, agricultural activity is on a decline, everyone is now buying and eating food items in rural areas. People do not engage in hard agricultural practices as they used to do. People now eat too much of unhealthy products." (ID: 55; Rural Morang; Health Facility Management Committee member)

One of the key stakeholders explained that despite ~~poor~~unhealthy dietary practice and physical inactivity being widespread, vulnerable group were disproportionately affected in terms of health effect.

"There are certain things about poor population as they have unhealthy diet but they are involved in lots of physical activity during digging, working; the disease differs between the poor and the rich. Physical activity is the good aspect among the poor but there are many other factors that are negative like unhealthy diet (consume more fast foods), inaccessibility of treatment facility and many stress factors among the poor people results in their diseased condition. Beside that behaviour of smoking and alcohol consumption among poor is another cause of disease." (Policy Stakeholder; ID: 4; Health)

CLD of major sub-systems depicting the interaction of the social determinants of ~~poor~~unhealthy diet and physical inactivity and NCDs

The CLDs below have been derived from the thematic analysis and show the dynamic interaction of the key variables discussed above contributing to the problem of NCDs in Nepal. As indicated, the development of the CLDs was an iterative process and was conducted simultaneously with thematic analysis. The CLDs depicts three prominent

feedback mechanisms or sub-systems displaying the interactions of the social determinants of NCDs: prevention delay, demand-supply and socio-economic influence.

In Fig 3~~figure 3~~, the prevention delay sub-system consists of delayed balancing loops, which highlight themes that indicate limited and delayed action by the health system towards addressing ~~poor~~unhealthy diet and physical inactivity-related practices as indicated in the results. Awareness-raising campaigns and multi-sectoral coordination for promoting sustainable food systems and physical activity-friendly environments were lacking for prevention of NCDs. The health system was possibly guided by the mental model that ~~poor~~unhealthy diet and physical inactivity were more due to individual behaviour, hence the interventions in developing countries were limited to campaigns and medical services provision. The CLD highlighted the themes indicating that multi-sectoral coordination for sustainable food systems and improving physical infrastructure were continually neglected by the overall health and social system. The themes also indicated that junk food companies are using various tactics to promote their products as a healthier option. The same sub-system hints at the curative orientation of the health system (i.e. the increasing chronic diseases problem in turn put pressure on the government to provide curative care services, leaving limited resources for preventative actions, including multi-sectoral coordination).

Fig 3. Prevention delay sub-system showing the interaction of governmental response to promote healthy diet and physical activity, other sub-systems and NCDs

Demand-supply sub-system (F~~figigure~~ 4) highlights how individuals and communities were being framed for consuming junk food and drinks due to limited policy and programmatic interventions relating to junk food/drink. The demand-supply sub-system mainly comprised of reinforcing loops and showed that junk food availability was resulting in increased consumption and affecting local food systems as well as social practices in the case district of Nepal. The extensive marketing of such products as healthy, easy to prepare, cost-effective, family food, etc. have driven the consumption of junk food. The junk food companies are possibly utilising their economic strength to influence both government and public through policy influences and marketing as indicated by study participants.

Fig 4. Demand-supply sub-system showing the interaction of availability and sales of junk food, other sub-systems and NCDs

Socio-economic sub-system (Fig~~figure~~ 5) can be conceptualised to distally drive the other two sub-systems towards increasing the burden of NCDs as per the thematic analysis. The sub-system shows the dynamic complexity about how junk foods, physical inactivity and social and economic influences were interacting, which was giving rise to NCDs problems. In particular, socio-economic situation was fuelling the migration of vulnerable groups from rural to city areas and foreign countries, which was not only pushing the rural agriculture system to the verge of collapse but also exposing rural people to unhealthy lifestyles. The sub-system also shows the disproportionate vulnerability and health effects experienced by the female members of migrant families and female in general.

Fig 5. Socio-economic influence sub-system showing the interaction of socio-economic status, other sub-systems for dietary and physical activity practices and NCDs

Discussion

The causal ~~mechanism of influence~~association of the diet and physical activity ~~on~~ with NCDs was complex and is discussed here in the light of existing evidence and from a systems perspective. Specifically, we utilise system archetypes (F~~f~~ig~~ure~~ 6), a simpler version of CLDs, to present the mechanism in a way that generates insights for health system action [34].

Fig 6 System archetypes showing different mechanisms of influence of dietary and physical activity practices on NCDs

Promotion of healthy dietary behaviour and physical activity

In this study, prevention-delay sub-system indicated that the health system has not been structured and functioning effectively to promote healthy behaviours relating to diet and physical activity. The health system in many developing countries like Nepal is yet to prioritise the prevention of NCDs and their behavioural risks [35-38]. In Nepal, evidence indicates that NCDs have been considered as a curative service agenda with increasing budgetary provisions in hospitals, patient care and development of clinical human resources rather than prevention [39]. This resonates with the *Fixes that Fail* system archetype (Fig~~figure~~ 6~~ ~~A).

Further, in many developing countries, national strategies for improving diet and physical activity exist [40-43] but most strategies are not effectively implemented due to limited resources and system inefficiency [44, 45]. In particular, developing countries are often lagging behind in terms of formulating and implementing legislations and programmes relating to control of junk food as well as developing physical activity-friendly infrastructures [20, 46], similar to the findings in this study. While developed countries are gradually addressing the junk food epidemic through policy measures, developing countries do not have any mechanism in place to control the junk food epidemic. The junk food companies are being increasingly compared to tobacco/alcohol companies due to their marketing strategies that target vulnerable children and population groups and their role in affecting the local food systems and ultimately health [20]. Further, poor systematic infrastructure-related policy action has been contributing to ~~poor~~ inadequate physical activity around the world [46, 47]. Prevention delay sub-system has illustrated that disease prevention and health promotion efforts in Nepal have been offset by systemic issues within health system. As a result, complex problems like NCDs continue to persist and there is tendency of health system to focus on easily implementable solutions than a comprehensive approach which require health system strengthening. This can be captured by the *Shifting Burden* archetype (Fig~~figure~~ 6~~-~~B). Many developing countries face similar health system challenges such as poor governance and management, resulting in inefficiency and ineffectiveness [48-51]. The health system continues to be ineffective and inefficient due to top-down approach, and leadership and management issues. Public health actions in developing countries are often short-sighted and implemented without collaborating effectively with other sectors. Strong leadership, appropriate system structure and political commitment could play a significant role in strengthening the health system and supporting comprehensive prevention initiatives in developing countries like Nepal [45, 52]. This shift will ensure more time and resources for the implementation of the prevention initiatives that is essential for combating complex problems like NCDs and their behavioural risks [53, 54].

Junk food access and marketing influencing consumption of junk foods

Demand-supply subsystem showed an increased access to highly processed food by everyone, especially young people. Evidence suggests that the junk food habit is very pervasive among children and adults in both urban and rural areas of South Asia region [55-58]. Further, a key mechanism that was prominent in the findings was the deskilling of the

traditional food preparation due to wider availability of the junk food and fast food. Evidence from around the world has also confirmed that traditional-based diets are gradually being replaced by junk food habit [59-62]. A review has highlighted how the local food systems were being shaped more by profit motives of the companies than by optimal nutrition [63]. In addition, migration of male youths, especially from rural areas, was driving the migration of families to urban areas, which is suddenly exposing families to an obesogenic environment.

Marketing of junk food is another key reason for the increasing access of the junk food as shown by different types of evidence [64] and highlighted by demand-supply sub-system. Often, the junk food companies through quality advertisements would associate their products with some social services or claim their products to be nutritious and can be enjoyed by an entire family [65]. Such practices of social marketing and misleading vulnerable population has been well documented in developed and developing countries [65-67]. Similar to the findings of this study, a report identified the price factor, easy availability and lack of government control were some of the factors that were leading to families providing children with junk food [65].

Enforcement agencies have limited ability to control junk food due to lack of regulations and positive framing of junk food by companies producing junk food. Junk food and sugar-sweetened beverages issues have not received much attention in public health policies and action in developing countries [20], which has also been reflected in the themes of this paper. Further, the effects of junk foods on local food systems have not been explored extensively. Developed countries with ample evidence of the effects of junk food are still facing stiff challenges in preventing the junk food induced obesity epidemic [68, 69]. The labelling of products is still a contested issue in Australia and New Zealand [70]. A Lancet review suggested that tactics of food companies influencing national policies can be considered similar to that of tobacco companies [20]. The *Drifting Goal* system archetype depicts the influence of food industries in delaying public health regulations for junk food such as sugar tax (Figfigure 6-C).

State of physical activity including traditional exercises

As noted in the themes and indicated in the CLD (interaction between all three subsystems), physical activity was on a decline in both urban and rural areas. A study in India showed that physical activity was gradually decreasing in rural India as well due to increasing urban

influences, including adopting urban lifestyle in rural settings, motor vehicle access and mechanisation of agricultural activities in rural areas [71]. Research in rural India and Bangladesh show a similar increasing phenomenon of physical inactivity in rural areas [72-74]. This study showed a shift in social practices (such as travelling, working and exercise practices) resulting in adults being physically inactive, which was further complicated by habit of consuming energy-dense food. A similar shift in diet and physical activity has been observed elsewhere in developing countries contributing to NCDs [75-77]. People only resorted to healthy diet and exercise after being affected with metabolic risk, a tendency that can be explained by behavioural and psychological theories [78, 79]. Evidence from South Asia region evidence has even suggested that people do not readily change their lifestyle even after being affected with NCDs metabolic risks [80].

The study findings suggested that traditional exercises were being ignored, which have potential to keep the general population healthy, based on cultural values and beliefs. The Vedic culture of Nepal (relating to Hindu religion) is rich in such exercise practices, particularly in yoga and meditations. Yoga is becoming increasingly popular in the Western countries due to its benefits [81] but continues to be neglected in Nepal. This can be explained by *Shifting the Burden* archetype (Fig~~figure~~ 6~~ ~~D) where the health system has not realised the most effective and preventive measures are based on local socio-cultural practices.

Socio-economic situation, migration and gender effect on dietary practice and physical activity

This study showed that socio-economic factors was influencing the diet and physical activity practices in multiple ways within the case districts. In rural areas, there was limited awareness about diet and physical activity. A relatively higher level of physical activity in rural areas could be attributed to agriculture and farming practices, while in urban areas, despite awareness, practice of balanced diet and physical activity was lacking. Available evidence also indicated this complex relationship [21, 82]. Some evidence showed that higher income groups were physically inactive while in some context, lower income groups had ~~poor~~ inadequate physical activity [83, 84]. A Lancet article noted this complexity and indicated that there may be a social patterning, which was shifting in the developing economy [21]. Such a shift of social patterning needs to be further researched. In this study, health was secondary to the vulnerable group who were experiencing economic hardship. More and

more people are migrating to urban areas due to economic hardship and unequal development, which is exposing people to urban influences of junk food diet and limited physical activity. A study in the US has shown that low-income households have a fatalistic attitude towards their health, possibly due to socio-economic circumstances and lack of ability to control their lives [85].

This study also found an increasing issue of ~~poor~~unhealthy diet and physical inactivity among women, both in urban and rural areas. While evidence to support our findings from rural areas was limited, but evidence suggest women in urban areas are more vulnerable to the obesogenic environment than male [57]. Urban females' lack of exercise could be understood by the fact that they remain mostly inactive due to confinement to household duties and child rearing, an outcome of Nepalese patriarchal social construct. A sense of the low power of females can be interpreted by limited access, time and autonomy for females to care for their health and engage in active lifestyles. Similar findings of limited autonomy among women relating to diet and physical activity related decisions have been reported elsewhere [86]. This study, particularly, identified the increased health vulnerability of rural women who migrated to urban areas due to sudden exposed to urban lifestyle. A more critical observation is needed to understand the level of autonomy and access to services of the women among different socio-economic group in both rural and urban areas.

The study findings indicated that the rapid migration and resulting decline in rural agricultural activity had both health and economic implications. The migration of economically active youths and their families to cities and foreign countries not only affected their lifestyle but also affect the local food systems and economy. A review has suggested that locally grown fresh foods were being replaced by imported vegetables and international junk food and supplies, which are cheaper [87]. As a result, the share of agriculture in the gross domestic product has been rapidly decreasing despite a significant (but declining) population still being engaged in agriculture and availability of improved technologies for the agriculture sector [88].

Further, the interaction of the socio-economic sub-system with the prevention delay sub-system (as shown in CLDs) is yet to be appreciated by the health system, thus resulting in limited action on social determinants of NCDs. This continuous ignorance of socio-economic determinants of NCDs by the health system closely resonates with *Shifting the Burden*

archetype (Fig~~figure~~ 6~~-~~E). This CLD model and its archetypes could provide critical insights to health system in addressing NCDs through comprehensive multi-sectoral action to address those socio-economic determinants of ~~poor~~unhealthy diet and physical inactivity. Health sector are in a good position to advocate and accelerate actions on the social determinants of NCDs in relation to diet and physical activity and these CLDs and archetypes could be a starting point for action.

~~**Limitations of the study**~~

The research comprised of some key limitations. The broader scope of the study framework meant that we could only partially illustrate the key social determinants of ~~poor~~unhealthy diet and physical inactivity risks in the context of Nepal. We have indicated within the paper that localised qualitative and quantitative studies are needed to understand the dynamics interactions of the social determinants of NCDs and their key risks factors. Further, the CLDs can be improved as new evidence emerges, which is one of the key strength of systems thinking approach to understand and tackle a complex problem. A key methodological challenge was limited participation of local stakeholders during the sense-making workshops, which did affect the quality of sense-making workshop.

Conclusion

This paper has presented a combination of key social determinants influencing dietary practices and physical activity in Nepal. Health system actions were delayed in terms of regulating junk food sales and coordinating with other sector for improving physical activity. While diet and physical activity-related knowledge and practices as well as environmental factors were poor, the broader determinants such as socio-economic status accelerated the migration to urban areas and exposure of population (in particular, female and children) to an obesogenic environment. In particular, traditional dietary and physical activity practices were being displaced negatively affecting one's health. Decline in agricultural activities was affecting physical activity level, local food systems and ultimately economy. The health system could play a key role in raising awareness, monitoring the markets for unethical marketing of junk food, and coordinating with other sectors for strengthening local food systems, creating infrastructure that promotes physical activity and addressing socio-economic determinants of ~~poor~~unhealthy diet and physical inactivity. ~~MoH~~ Ministry of Health could advocate and show evidence of the need for such preventative action through

the creation of a powerful national agency to facilitate inter-sectoral collaboration and achieve SDH focus.

Acknowledgement

The paper is derived from the PhD project of the first author and hence would like to acknowledge all the participants and supporters of the PhD study including Ministry of Health/Nepal, District Public Health Office Morang, District Public Health Office Bhaktapur. The authors would also like to thank research ethics committees of Massey University and Nepal Health Research Council. We would like to acknowledge Mr. Shiva Raj Mishra (PhD Student at University of Queensland) and Mr. Mohan Paudel (PhD Student at Flinders University) for their critical review and suggestions on this paper.

References

1. World Health Organization. Global Status Report on noncommunicable diseases 2014. World Health Organization, 2014.
2. Lachat C, Otchere S, Roberfroid D, Abdulai A, Seret FMA, Milesevic J, et al. Diet and Physical Activity for the Prevention of Noncommunicable Diseases in Low- and Middle-Income Countries: A Systematic Policy Review. PLOS Medicine. 2013;10(6):e1001465. doi: 10.1371/journal.pmed.1001465.
3. Lee IM, Shiroma EJ, Lobelo F, Puska P, Blair SN, Katzmarzyk PT. Impact of Physical Inactivity on the World's Major Non-Communicable Diseases. Lancet. 2012;380(9838):219-29. doi: 10.1016/S0140-6736(12)61031-9. PubMed PMID: PMC3645500.
4. Aryal KK, Mehata S, Neupane S, Vaidya A, Dhimal M, Dhakal P, et al. The Burden and Determinants of Non Communicable Diseases Risk Factors in Nepal: Findings from a Nationwide STEPS Survey. PloS one. 2015;10(8):e0134834. Epub 2015/08/06. doi: 10.1371/journal.pone.0134834. PubMed PMID: 26244512; PubMed Central PMCID: PMCPMC4526223.
5. Dhungana RR, Bista B, Pandey AR, de Courten M. Prevalence, clustering and sociodemographic distributions of non-communicable disease risk factors in Nepalese adolescents: secondary analysis of a nationwide school survey. BMJ open. 2019;9(5):e028263.
6. Kohl HW, 3rd, Craig CL, Lambert EV, Inoue S, Alkandari JR, Leetongin G, et al. The pandemic of physical inactivity: global action for public health. The Lancet. 380(9838):294-305. doi: 10.1016/S0140-6736(12)60898-8.
7. Graham H, White PCL. Social determinants and lifestyles: integrating environmental and public health perspectives. Public health. 2016;141:270-8. doi: https://doi.org/10.1016/j.puhe.2016.09.019.
8. World Health Organization. United Nations high-level meeting on noncommunicable disease prevention and control: NCD summit to shape the international agenda. New York, NY: Author; 2011 [cited 2013 May 15].

Formatted: Indent: Left: 0", Hanging: 0.39"

Formatted: Font: (Default) Times New Roman, 12 pt

Formatted: Font: (Default) Times New Roman, 12 pt

Formatted: Font: (Default) Times New Roman, 12 pt

9. World Health Organization. Rio Political Declaration on Social Determinants of Health, Rio de Janeiro, Brazil. 2011. 2011.
10. World Health Organization. Sustainable Development Goals 2015 [cited 2015]. Available from: https://sustainabledevelopment.un.org/sdgs.
11. Solar O, Irwin A. A conceptual framework for action on the social determinants of health. Geneva: World Health Organization, 2010.
12. Marmot M, Bell R. Social determinants and non-communicable diseases: time for integrated action. Bmj. 2019;364:l251. doi: 10.1136/bmj.l251.
13. Donkin A, Goldblatt P, Allen J, Nathanson V, Marmot M. Global action on the social determinants of health. BMJ global health. 2017;3(Suppl 1):e000603-e. doi: 10.1136/bmjgh-2017-000603. PubMed PMID: 29379648.
14. Marmot M, Allen J, Goldblatt P, Boyce T, McNeish D, Grady M, et al. The Marmot review: Fair society, healthy lives. The Strategic Review of Health Inequalities in England Post-2010. 2010.
15. National Advisory Committee. The social, cultural and economic determinants of health in New Zealand: Action to improve health. Wellington: National Advisory Committee. 1998.
16. Marmot M, Allen J, Bell R, Bloomer E, Goldblatt P. WHO European review of social determinants of health and the health divide. The lancet. 2012;380(9846):1011-29.
17. Ball K, Carver A, Downing K, Jackson M, O'Rourke K. Addressing the social determinants of inequities in physical activity and sedentary behaviours. Health promotion international. 2015;30(suppl_2):ii8-ii19.
18. Fortune K, de Snyder NS, Galvão LAC, Murphy M. The Social Determinants of NCDs. Economic Dimensions of Noncommunicable Diseases in Latin America and the Caribbean. 2016:31.
19. Villalobos Dintrans P, Rodriguez L, Clingham-David J, Pizarro T. Implementing a Food Labeling and Marketing Law in Chile. Health Systems & Reform. 2020;6(1):e1753159. doi: 10.1080/23288604.2020.1753159.
20. Moodie R, Stuckler D, Monteiro C, Sheron N, Neal B, Thamarangsi T, et al. Profits and pandemics: prevention of harmful effects of tobacco, alcohol, and ultra-processed food and drink industries. The Lancet. 2013;381(9867):670-9.
21. Hallal PC, Andersen LB, Bull FC, Guthold R, Haskell W, Ekelund U. Global physical activity levels: surveillance progress, pitfalls, and prospects. The Lancet. 2012;380(9838):247-57. doi: 10.1016/S0140-6736(12)60646-1.
22. Singh DR, Sunuwar DR, Dahal B, Sah RK. The association of sleep problem, dietary habits and physical activity with weight status of adolescents in Nepal. BMC public health. 2021;21(1):938. doi: 10.1186/s12889-021-10985-5.
23. Paudel S, Owen AJ, Smith BJ. Exploration of Physical Activity Barriers and Facilitators Among Adults in Kathmandu, Nepal. Qualitative Health Research. 2021;31(6):1183-95. doi: 10.1177/1049732321993096.
24. Oli N, Vaidya A, Pahkala K, Eiben G, Krettek A. Knowledge, attitude and practice on diet and physical activity among mothers with young children in the Jhaukhel-Duwakot Health Demographic Surveillance Site, Nepal. PloS one. 2018;13(7):e0200329. doi: 10.1371/journal.pone.0200329.
25. Das P, Horton R. Physical activity—time to take it seriously and regularly. The Lancet. 388(10051):1254-5. doi: 10.1016/S0140-6736(16)31070-4.
26. Midgley G. Systemic Intervention for Public Health. American journal of public health. 2006;96(3):466-72. doi: 10.2105/AJPH.2005.067660. PubMed PMID: PMC1470519.
27. Sharma SR, Matheson A, Lambrick D, Faulkner J, Lounsbury DW, Vaidya A, et al. The role of tobacco and alcohol use in the interaction of social determinants of non-

Formatted: Font: (Default) Times New Roman, 12 pt

Formatted: Font: (Default) Times New Roman, 12 pt

communicable diseases in Nepal: a systems perspective. BMC public health. 2020;20(1):1368. doi: 10.1186/s12889-020-09446-2.

28. Palinkas LA, Horwitz SM, Green CA, Wisdom JP, Duan N, Hoagwood K. Purposeful sampling for qualitative data collection and analysis in mixed method implementation research. Administration and Policy in Mental Health and Mental Health Services Research. 2015;42(5):533-44.
29. Smith J, Firth J. Qualitative data analysis: the framework approach. Nurse researcher. 2011;18(2):52-62.
30. Sterman JD. Business dynamics: Systems thinking and modeling for a complex world. Boston: Irwin/McGraw-Hill; 2000.
31. Microsoft. System requirements for Office. Available from: https://products.office.com/en-nz/office-system-requirements#coreui-contentrichblock-nnuz9v1.
32. Dedoose. Home 2016. Available from: http://www.dedoose.com/.
33. Ventana Systems Inc. Vensim Home USA: Ventana Systems, Inc.; 2015. Available from: http://vensim.com/.
34. Kim DH. Systems archetypes I: diagnosing systemic issues and designing high-leverage interventions: Pegasus Communications; 1993.
35. Department of Health Services. Annual Report 2015/16. Kathmandu, Nepal: Ministry of Health, 2016.
36. Department of Health and Family Welfare. Annual Report 2015/16. India: Ministry of Health and Family Welfare, 2016.
37. Medical Statistics Unit. Annual Health Bulletin. India: Ministry of Health, Nutrition and Indigenous Medicine, 2015.
38. Horton R. Offline: NCDs - why are we failing? The Lancet. 390(10092):346. doi: 10.1016/S0140-6736(17)31919-0.
39. Ministry of Finance. Budget Speech 2071/72 Nepal Kathmandu: Government of Nepal; 2015. Available from: http://mof.gov.np/uploads/cmsfiles/file/budget%20speech%202071-72_20140713125927.pdf.
40. Ministry of Health and Family Welfare. National action plan and monitoring framework for prevention and control of noncommunicable disease (NCDs) in India. Government of India; 2013.
41. MInistry of Health. NCD Action Plan Brazil: 2011-2022. Government of Brazil; 2011.
42. MInistry of Health. National Policy for the prevention and control of chronic non-communicable diseases in Ghana. Government of Ghana; 2012.
43. Ministry of Healthcare and Nutrition. The national policy and strategic framework for prevention and control of chronic non-communicable diseases. Government of Srilanka; 2009.
44. World Health Organization. Noncommunicable diseases progress monitor 2015. 2015.
45. Sharma SR, Page R, Matheson A, Lambrick D, Faulkner J, Mishra SR. Non-communicable disease prevention in Nepal: systemic challenges and future directions. Global health promotion. 2017:1757975917720800. doi: 10.1177/1757975917720800.
46. Reis RS, Salvo D, Ogilvie D, Lambert EV, Goenka S, Brownson RC, et al. Scaling up physical activity interventions worldwide: stepping up to larger and smarter approaches to get people moving. The lancet. 2016;388(10051):1337-48.
47. Sallis JF, Cerin E, Conway TL, Adams MA, Frank LD, Pratt M, et al. Physical activity in relation to urban environments in 14 cities worldwide: a cross-sectional study. The Lancet. 387(10034):2207-17. doi: https://doi.org/10.1016/S0140-6736(15)01284-2.

Formatted: Font: (Default) Times New Roman, 12 pt
Formatted: Font: (Default) Times New Roman, 12 pt
Formatted: Font: (Default) Times New Roman, 12 pt
Formatted: Font: (Default) Times New Roman, 12 pt
Formatted: Font: (Default) Times New Roman, 12 pt
Formatted: Font: (Default) Times New Roman, 12 pt
Formatted: Font: (Default) Times New Roman, 12 pt
Formatted: Font: (Default) Times New Roman, 12 pt

48. Ahmad B, Fouad FM, Elias M, Zaman S, Phillimore P, Maziak W. Health system challenges for the management of cardiovascular disease and diabetes: an empirical qualitative study from Syria. International journal of public health. 2015;60(1):55-62. doi: 10.1007/s00038-014-0594-2.
49. Willis-Shattuck M, Bidwell P, Thomas S, Wyness L, Blaauw D, Ditlopo P. Motivation and retention of health workers in developing countries: a systematic review. BMC health services research. 2008;8(1):247. doi: 10.1186/1472-6963-8-247.
50. Marten R, McIntyre D, Travassos C, Shishkin S, Longde W, Reddy S, et al. An assessment of progress towards universal health coverage in Brazil, Russia, India, China, and South Africa (BRICS). The Lancet. 2014;384(9960):2164-71. doi: https://doi.org/10.1016/S0140-6736(14)60075-1.
51. Balarajan Y, Selvaraj S, Subramanian SV. Health care and equity in India. The Lancet. 2011;377(9764):505-15. doi: https://doi.org/10.1016/S0140-6736(10)61894-6.
52. Alleyne G, Binagwaho A, Haines A, Jahan S, Nugent R, Rojhani A, et al. Embedding non-communicable diseases in the post-2015 development agenda. The Lancet. 2013;381(9866):566-74.
53. Bonita R, Magnusson R, Bovet P, Zhao D, Malta DC, Geneau R, et al. Country actions to meet UN commitments on non-communicable diseases: a stepwise approach. The Lancet. 2013;381(9866):575-84.
54. Beaglehole R, Ebrahim S, Reddy S, Voûte J, Leeder S. Prevention of chronic diseases: a call to action. The Lancet. 2008;370(9605):2152-7.
55. Misra A, Singhal N, Khurana L. Obesity, the metabolic syndrome, and type 2 diabetes in developing countries: role of dietary fats and oils. Journal of the American College of Nutrition. 2010;29(3 Suppl):289s-301s. Epub 2010/09/18. PubMed PMID: 20823489.
56. Pradeepa R, Anjana RM, Joshi SR, Bhansali A, Deepa M, Joshi PP, et al. Prevalence of generalized & abdominal obesity in urban & rural India- the ICMR - INDIAB Study (Phase-I) [ICMR - INDIAB-3]. The Indian journal of medical research. 2015;142(2):139-50. doi: 10.4103/0971-5916.164234. PubMed PMID: PMC4613435.
57. Vaidya A, Shakya S, Krettek A. Obesity Prevalence in Nepal: Public Health Challenges in a Low-Income Nation during an Alarming Worldwide Trend. International journal of environmental research and public health. 2010;7(6):2726-44. doi: 10.3390/ijerph7062726. PubMed PMID: PMC2905575.
58. Ramachandran A, Snehalatha C. Rising burden of obesity in Asia. Journal of obesity. 2010;2010.
59. Waqanivalu TK. Pacific islanders pay heavy price for abandoning traditional diet. Bulletin of the World Health Organization. 2010;88(7):484-5.
60. Kolahdooz F, Sheikholeslam R, Naghavi M, Abdollahi Z. Junk food consumption: an indicator of changing dietary habit in Iranian children. Asia Pacific journal of clinical nutrition. 2004;13.
61. Hassan Wassef H. Food habits of the Egyptians: newly emerging trends. 2004.
62. Jacobs A, Richtel M. How big business got Brazil hooked on junk food. New York Times, Sept. 2017;16:2017.
63. Stuckler D, Nestle M. Big Food, Food Systems, and Global Health. PLOS Medicine. 2012;9(6):e1001242. doi: 10.1371/journal.pmed.1001242.
64. Sadeghirad B, Duhaney T, Motaghipisheh S, Campbell NRC, Johnston BC. Influence of unhealthy food and beverage marketing on children's dietary intake and preference: a systematic review and meta- analysis of randomized trials. Obesity Reviews. 2016;17(10):945-59. doi: doi:10.1111/obr.12445.

Formatted: Font: (Default) Times New Roman, 12 pt

Formatted: Font: (Default) Times New Roman, 12 pt

Formatted: Font: (Default) Times New Roman, 12 pt

Formatted: Font: (Default) Times New Roman, 12 pt

65. RECPHEC. Rapid assessment on media coverage of junk food and its content analysis on selected Nepali and Indian Television Channels. Kathmandu, Nepal: Resource Centre for Primary Health Care (RECPHEC), 2013.
66. Dixon HG, Scully ML, Wakefield MA, White VM, Crawford DA. The effects of television advertisements for junk food versus nutritious food on children's food attitudes and preferences. Social Science & Medicine. 2007;65(7):1311-23. doi: https://doi.org/10.1016/j.socscimed.2007.05.011.
67. Hastings G, McDermott L, Angus K, Stead M, Thomson S. The extent, nature and effects of food promotion to children: a review of the evidence. Geneva: World Health Organization. 2006;20.
68. Nestle M. Food politics: How the food industry influences nutrition and health: Univ of California Press; 2013.
69. Jolly R. Marketing Obesity?: Junk Food, Advertising and Kids: Parliamentary Library; 2011.
70. Magnusson RS. Obesity prevention and personal responsibility: the case of front-of-pack food labelling in Australia. BMC public health. 2010;10(1):662. doi: 10.1186/1471-2458-10-662.
71. Tripathy JP, Thakur JS, Jeet G, Chawla S, Jain S, Prasad R. Urban rural differences in diet, physical activity and obesity in India: are we witnessing the great Indian equalisation? Results from a cross-sectional STEPS survey. BMC public health. 2016;16(1):816. doi: 10.1186/s12889-016-3489-8.
72. Anjana RM, Pradeepa R, Das AK, Deepa M, Bhansali A, Joshi SR, et al. Physical activity and inactivity patterns in India – results from the ICMR-INDIAB study (Phase-1) [ICMR-INDIAB-5]. The international journal of behavioral nutrition and physical activity. 2014;11:26-. doi: 10.1186/1479-5868-11-26. PubMed PMID: PMC3974063.
73. Moniruzzaman M, Zaman MM, Islalm M, Ahasan H, Kabir H, Yasmin R. Physical activity levels in Bangladeshi adults: results from STEPS survey 2010. Public health. 2016;137:131-8.
74. Ng N, Hakimi M, Van Minh H, Juvekar S, Razzaque A, Ashraf A, et al. Prevalence of physical inactivity in nine rural INDEPTH Health and Demographic Surveillance Systems in five Asian countries. Global health action. 2009;2(1):1985.
75. Kimokoti RW, Fung TT, Millen BE. Chapter 7 - Dietary Patterns/Diet and Health of Adults in Economically Developing Countries. Bioactive Food as Dietary Interventions for the Aging Population. San Diego: Academic Press; 2013. p. 83-107.
76. Pou SA, del Pilar Díaz M, De La Quintana AG, Forte CA, Aballay LR. Identification of dietary patterns in urban population of Argentina: study on diet-obesity relation in population-based prevalence study. Nutrition Research and Practice. 2016;10(6):616-22. doi: 10.4162/nrp.2016.10.6.616. PubMed PMID: PMC5126411.
77. Popkin BM, Adair LS, Ng SW. NOW AND THEN: The Global Nutrition Transition: The Pandemic of Obesity in Developing Countries. Nutrition reviews. 2012;70(1):3-21. doi: 10.1111/j.1753-4887.2011.00456.x. PubMed PMID: PMC3257829.
78. Prochaska JO, Velicer WF. The transtheoretical model of health behavior change. American journal of health promotion. 1997;12(1):38-48.
79. Scharff RL. Obesity and hyperbolic discounting: Evidence and implications. Journal of Consumer Policy. 2009;32(1):3-21.
80. Lawton J, Ahmad N, Hanna L, Douglas M, Hallowell N. 'I can't do any serious exercise': barriers to physical activity amongst people of Pakistani and Indian origin with Type 2 diabetes. Health education research. 2006;21(1):43-54. doi: 10.1093/her/cyh042.
81. Harvard Health Publishing. An Introduction to Yoga. Harvard Medical School, 2016.

Formatted: Font: (Default) Times New Roman, 12 pt

Formatted: Font: (Default) Times New Roman, 12 pt

82. Hallal PC, Matsudo SM, Matsudo VKR, Araújo TL, Andrade DR, Bertoldi AD. Physical activity in adults from two Brazilian areas: similarities and differences. Cadernos de saude publica. 2005;21:573-80.
83. Knuth AG, Bacchieri G, Victora CG, Hallal PC. Changes in physical activity among Brazilian adults over a 5-year period. J Epidemiol Community Health. 2010;64(7):591-5. Epub 2009/08/27. doi: 10.1136/jech.2009.088526. PubMed PMID: 19706621.
84. Singh A, Purohit B. Physical activity, sedentary lifestyle, and obesity among Indian dental professionals. Journal of Physical Activity and Health. 2012;9(4):563-70.
85. Keeley B, Wright L, Condit CM. Functions of health fatalism: fatalistic talk as face saving, uncertainty management, stress relief and sense making. Sociology of health & illness. 2009;31(5):734-47.
86. Balarajan Y, Villamor E. Nationally representative surveys show recent increases in the prevalence of overweight and obesity among women of reproductive age in Bangladesh, Nepal, and India. The Journal of nutrition. 2009;139(11):2139-44. Epub 2009/09/25. doi: 10.3945/jn.109.112029. PubMed PMID: 19776182.
87. Bishwajit G. Nutrition transition in South Asia: the emergence of non-communicable chronic diseases. F1000Research. 2015;4:8. doi: 10.12688/f1000research.5732.2. PubMed PMID: PMC4706051.
88. World Bank. Data: Agriculture, forestry, and fishing, value added (% of GDP) 2018. Available from: https://data.worldbank.org/indicator/NV.AGR.TOTL.ZS.

Formatted: Font: (Default) Times New Roman, 12 pt

Formatted: Font: (Default) Times New Roman, 12 pt

Supporting information

S1 File. COREC Checklist

S2 File. Interview and FGD guidelines

Response to reviewers

1. Please ensure that your manuscript meets PLOS ONE's style requirements, including those for file naming. The PLOS ONE style templates can be found at

https://journals.plos.org/plosone/s/file?id=wjVg/PLOSOne_formatting_sample_main_body.pdf and https://journals.plos.org/plosone/s/file?id=ba62/PLOSOne_formatting_sample_title_authors_affiliations.pdf

- Thank you for your suggestion. We have reviewed and reformatted the manuscript as per the PLOS ONE style.

2. We noted in your submission details that a portion of your manuscript may have been presented or published elsewhere. [This article is different from a related published (https://bmcpublichealth.biomedcentral.com/articles/10.1186/s12889-020-09446-2) article in terms of findings and discussion. Since the article is based on my PhD thesis, articles that have been published (or will be published) have similar methods section (and hence the use of same figure relating to methodology and study framework).] Please clarify whether this [conference proceeding or publication] was peer-reviewed and formally published. If this work was previously peer-reviewed and published, in the cover letter please provide the reason that this work does not constitute dual publication and should be included in the current manuscript.

- This is the second paper from the PhD thesis and includes results about diet and physical activity. The first paper concentrated on results from the PhD focussing on tobacco and alcohol use (https://bmcpublichealth.biomedcentral.com/articles/10.1186/s12889-020-09446-2) which has been peer-reviewed and published. Since the first published paper and this paper on diet and physical activity are from the first authors PhD thesis, we have used and referenced figures relating to the methods and study framework from the published paper in the submitted paper focusing on diet and physical activity. The introduction, findings and discussion are entirely different to the published paper and have not been published elsewhere and hence, we do not feel this constitutes dual publication.

.

3. PLOS requires an ORCID iD for the corresponding author in Editorial Manager on papers submitted after December 6th, 2016. Please ensure that you have an ORCID iD and that it is validated in Editorial Manager. To do this, go to 'Update my Information' (in the upper left-hand corner of the main menu), and click on the Fetch/Validate link next to the ORCID field. This will take you to the ORCID site and allow you to create a new iD or authenticate a pre-existing iD in Editorial Manager. Please see the following video for instructions on linking an ORCID iD to your Editorial Manager account: https://www.youtube.com/watch?v=_xcclfuvtxQ

- ORCID ID of the corresponding author (SS): https://orcid.org/0000-0002-7880-5517

4. Please review your reference list to ensure that it is complete and correct. If you have cited papers that have been retracted, please include the rationale for doing so in the manuscript text, or remove these references and replace them with relevant current references. Any changes to the reference list should be mentioned in the rebuttal letter that accompanies your revised manuscript. If you need to cite a retracted article, indicate the article's retracted status in the References list and also include a citation and full reference for the retraction notice.

- The reference list has been reviewed and is complete and correct.

[Note: HTML markup is below. Please do not edit.]

Reviewers' comments:

Reviewer's Responses to Questions

Comments to the Author

1. Is the manuscript technically sound, and do the data support the conclusions?

The manuscript must describe a technically sound piece of scientific research with data that supports the conclusions. Experiments must have been conducted rigorously, with appropriate controls, replication, and sample sizes. The conclusions must be drawn appropriately based on the data presented.

Reviewer #1: Partly

Reviewer #2: Yes

Thank you for considering our manuscript as scientifically and technically sound.

---

2. Has the statistical analysis been performed appropriately and rigorously?

Reviewer #1: Yes

Reviewer #2: N/A

We agree that statistical analysis is not applicable for this study.

---

3. Have the authors made all data underlying the findings in their manuscript fully available?

The PLOS Data policy requires authors to make all data underlying the findings described in their manuscript fully available without restriction, with rare exception (please refer to the

Data Availability Statement in the manuscript PDF file). The data should be provided as part of the manuscript or its supporting information, or deposited to a public repository. For example, in addition to summary statistics, the data points behind means, medians and variance measures should be available. If there are restrictions on publicly sharing data—e.g. participant privacy or use of data from a third party—those must be specified.

Reviewer #1: No

Reviewer #2: Yes

We agree with reviewer 1 that still there is limitation in data availability and have shared that with editors. We are in the process of making data available public once we review the transcripts and make sure all personal identifiers of the respondents are removed, as Nepal is a small country and participants from policy level are easily identified. We hope to make the data publicly available by this year.

____

4. Is the manuscript presented in an intelligible fashion and written in standard English?

PLOS ONE does not copyedit accepted manuscripts, so the language in submitted articles must be clear, correct, and unambiguous. Any typographical or grammatical errors should be corrected at revision, so please note any specific errors here.

Reviewer #1: Yes

Reviewer #2: Yes

Thank you.

____

5. Review Comments to the Author

Please use the space provided to explain your answers to the questions above. You may also include additional comments for the author, including concerns about dual publication, research ethics, or publication ethics. (Please upload your review as an attachment if it exceeds 20,000 characters)

Reviewer #1: This is a very interesting and mostly well-written paper. The authors utilize appropriate methods to draw their conclusions
Some minor comments
1. Please consider using sex instead of gender, given you did not record one's gender. In case you measured one's gender, this should be more clearly presented in the methods

- ***Thank you for your kind suggestion and we agree that we have not measured gender within this paper. However, gender reflects socially constructed characteristics of male and female. We have indeed provided some context such as Nepal being patriarchal society and having more social and economic power as a breadwinner of the family. Also, our findings and discussion are linking those gendered experiences with diet and physical activity related practices. Thus, we feel that it makes more sense if we keep the term "gender" within the manuscript.***

2. The manuscript requires some English editing

- ***The English has been reviewed by co-authors who are native English speakers.***

3. Avoid using terms such as "causal relationship", "influence" etc. Given your study design cannot support these claims, please replace with association etc

- ***Thank you for your kind suggestion. We have revised and replaced "causal relationship" with "association" accordingly. However, the term "Causal Loop Diagrams" is a standard method/tool name and we have not changed it. Also, as this is a qualitative study, keeping "influence(s)" made more sense (qualitative term) than replacing with "association" (quantitative term) and hence have decided to keep the term "influence (s)" as it is.***

Reviewer #2: Very timely and useful research. Probably, on of the most critical public health issue of our time is the growing burden of non-communicable disease. Most of these diseases are preventable and heavily dependent on behavior change. Changing people's behavior requires a thorough understanding of the root causes of behaviors and health system-related structural issue and the interlinkages with social-determinants of health leading to NCDs. The authors clearly described and organized their findings along these lines. Just two question.
1. Why were the study areas purposively selected? What was the rational for this judgment sampling rather than random selection? Was it to save time? I understand the application of the method to identify participants within the selected communities, as you stated because of their knowledge and experience. But, why were the communities selected through this method?

- ***This is a qualitative study and often, cases are purposively chosen. In the study, the main purpose was to choose different context i.e. urban, semi-urban and rural settings, and was also selected in consultation with District Health Offices. Time and convenience of the first author (principal investigator) also influenced the choice of districts which is acceptable in purposive sampling. In qualitative studies, often random sampling is not the choice of sampling.***

2. Most of the findings do not seem new. It would be helpful, if the authors emphasized on what is the key new knowledge versus what was already known. For example, we know urbanization, increasing availability and consumption of junk food, alcohol consumption, smoking and reduced physical activities are risk factors for NCDs. Is the key on the findings the Nepalese context?

- ***The findings are especially important in Nepalese context where the focus is on changing behaviour. Yes, the findings may not be new for high-income countries but, for low income countries like Nepal, the authors hope that this paper can provide a whole new way of seeing a public health issue, taking a systems perspective and designing appropriate actions. This paper provides qualitative evidence on social determinants of dietary and physical activity practices which is very scarce in Nepalese (or low income country) and we hope that this provides some systemic insights for actions by concerned health agencies in Nepal.***

---

6. PLOS authors have the option to publish the peer review history of their article (what does this mean?). If published, this will include your full peer review and any attached files.

If you choose "no", your identity will remain anonymous but your review may still be made public.

Do you want your identity to be public for this peer review? For information about this choice, including consent withdrawal, please see our Privacy Policy.

Reviewer #1: No

Reviewer #2: No

[NOTE: If reviewer comments were submitted as an attachment file, they will be attached to this email and accessible via the submission site. Please log into your account, locate the manuscript record, and check for the action link "View Attachments". If this link does not appear, there are no attachment files.]

While revising your submission, please upload your figure files to the Preflight Analysis and Conversion Engine (PACE) digital diagnostic tool, https://pacev2.apexcovantage.com/. PACE helps ensure that figures meet PLOS requirements. To use PACE, you must first register as a user. Registration is free. Then, login and navigate to the UPLOAD tab, where you will find detailed instructions on how to use the tool. If you encounter any issues or have any questions when using PACE, please email PLOS at figures@plos.org. Please note that Supporting Information files do not need this step.

In compliance with data protection regulations, you may request that we remove your personal registration details at any time. (Remove my information/details). Please contact the publication office if you have any questions.

Response to editors

We've checked your submission and before we can proceed, we need you to address the following issues:

1. It appears that your ORCiD iD has not been validated in your Editorial Manager account and we are unable to proceed until that step is complete.

To validate your ORCiD iD in Editorial Manager, please follow the steps below:

1.1 In your Editorial Manager account, please go to 'Update my Information' (in the upper left-hand corner of the main menu), and click on the Fetch/Validate link next to the ORCiD field.

1.2 This link will take you to the ORCiD site and allow you to create a new iD or authenticate a pre-existing iD in Editorial Manager.

For additional instructions, please watch the following video for a step-by-step demonstration: https://www.youtube.com/watch?v=_xcclfuvtxQ

- **Dear editors, I am not able to update my ORCID ID automatically in the Editorial Manager. It keeps saying "An identical, Validated ORCID already exists in the database. An ORCID may be linked to only one record in the database, and so you may have registered another user record in the past and retrieved/validated your ORCID for use with that."**

- **I checked if I had duplicate ID but I do not seem to have. I do not know the reason for the error. Please find my ORCID ID for your kind perusal and use: https://orcid.org/0000-0002-7880-5517**

2. We note your current data availability statement:

"Transcripts (without any personal identifier) and study tools are available on request (Email: yoursudesh@gmail.com; r.a.page@massey.ac.nz). This paper is part of the PhD study of the first author, and after completion of the PhD study, all transcripts will be available through an open access data repository."

Please note that PLOS only permits data to be made available on request if there are ethical or legal restrictions on sharing the data. Before we proceed, we'll require some additional information to ensure your submission adheres to the PLOS policy on acceptable data access restrictions: https://journals.plos.org/plosone/s/data-availability#loc-acceptable-data-access-restrictions.

1) Please confirm whether there are legal or ethical restrictions on sharing your data publicly.

- **Upon checking my University's Ethics Application and with supervisors, I can confirm that there are ethical restrictions for sharing my qualitative data publicly.**
- **As per my approved Ethics application, I am allowed to use quotes from the interviews but not able to upload the interviews in the official archive at least for 10 years - and only of those participants who consented to upload in public repository. Participants were specifically asked in the consent form if they are ok with making the interviews transcripts available in an official achieve. Most of the participants in the consent form have said "no" to uploading in public repository.**
- **Further, we are not sure about the idea that someone could use the qualitative data to try and replicate findings. Analysis of interviews is a subjective exercise.**
- **Please find the revised data availability statement:**

  "Transcripts (without any personal identifier and of those who consented to sharing of the interview transcripts) are available on request (Email: yoursudesh@gmail.com; r.a.page@massey.ac.nz) after signing confidentiality agreement due to ethical reasons. "

- **We have further added a condition of signing confidentiality agreement in the data availability statement if anyone request us the data. This is to ensure confidentiality of the stakeholders from Nepal who may be easily identifiable from the transcripts data.**

2) If legal or ethical restrictions apply, please provide all necessary instructions and non-author contact information (preferably email) for a data access committee, ethics committee, or other institutional body that other researchers would require to request access to your data.

Note that it is not acceptable for an author to be the sole named individual responsible for ensuring data access.

Please find the email address of all my supervisors who were consulted for the response.

- **As for the data access, we have a process. The primary author is the main contact point as a corresponding author for data access, who will forward the request to all supervisors for consideration. If we do not find any issue with the request collectively, the primary author will send a confidentiality agreement as well as verify the request as genuine. Please note that, we are able to share the data confidentially only of those participants who consented to sharing of the interview data.**
- **Here are the email addresses of my supervisors and Massey University Ethics Committee for your necessary action:**

Rachel Page: r.a.page@massey.ac.nz
Anna Matheson: anna.matheson@vuw.ac.nz
David Lounsbury: david.lounsbury@einsteinmed.org

James.Faulkner: James.Faulkner@winchester.ac.uk
Danielle Lambrick: D.M.Lambrick@soton.ac.uk
Abhinav Vaidya: dr.abhinavaidya@gmail.com
Massey's Human Ethics Committee: humanethics@massey.ac.nz (Human Ethics Application SOA 16/37)

3) If there are no legal or ethical restrictions on sharing your data publicly, please upload the minimal anonymized data set necessary to replicate your study findings as either Supporting Information files, or to a stable public repository. If you upload your data to a repository, please also provide the relevant URLs, DOIs, or accession numbers for other researchers to access your data directly. For a list of recommended public repositories, please see https://journals.plos.org/plosone/s/recommended-repositories.

- **As mentioned above, ethical restrictions apply**

- **Here is the updated Data availability statement:**

  "Transcripts (without any personal identifier and of those who consented to sharing of the interview transcripts) are available on request (Email: yoursudesh@gmail.com; r.a.page@massey.ac.nz) after signing a confidentiality agreement due to ethical reasons. "
