# Supplementary material for: Dietary practices, physical activity and social determinants of non-communicable diseases in Nepal: A systemic analysis
Source: PLoS One. 2023 Feb 6;18(2):e0281355. doi: 10.1371/journal.pone.0281355 (PMC9901760; doi:10.1371/journal.pone.0281355)
Supplement: Attachment — Submitted filename: 220511 Ethics response.docx [file pone.0281355.s003.docx]

# **Response to editors**

We've checked your submission and before we can proceed, we need you to address the following issues:

1. It appears that your ORCiD iD has not been validated in your Editorial Manager account and we are unable to proceed until that step is complete.

To validate your ORCiD iD in Editorial Manager, please follow the steps below:

1.1 In your Editorial Manager account, please go to ‘Update my Information’ (in the upper left-hand corner of the main menu), and click on the Fetch/Validate link next to the ORCiD field.

1.2 This link will take you to the ORCiD site and allow you to create a new iD or authenticate a pre-existing iD in Editorial Manager.

For additional instructions, please watch the following video for a step-by-step demonstration: <https://www.youtube.com/watch?v=_xcclfuvtxQ>

- **Dear editors, I am not able to update my ORCID ID automatically in the Editorial Manager. It keeps saying “An identical, Validated ORCID already exists in the database. An ORCID may be linked to only one record in the database, and so you may have registered another user record in the past and retrieved/validated your ORCID for use with that.”**
- **I checked if I had duplicate ID but I do not seem to have. I do not know the reason for the error. Please find my ORCID ID for your kind perusal and use:** [**https://orcid.org/0000-0002-7880-5517**](https://orcid.org/0000-0002-7880-5517)

2. We note your current data availability statement:

"Transcripts (without any personal identifier) and study tools are available on request (Email: [yoursudesh@gmail.com](mailto:yoursudesh@gmail.com); [r.a.page@massey.ac.nz](mailto:r.a.page@massey.ac.nz)). This paper is part of the PhD study of the first author, and after completion of the PhD study, all transcripts will be available through an open access data repository."

Please note that PLOS only permits data to be made available on request if there are ethical or legal restrictions on sharing the data. Before we proceed, we’ll require some additional information to ensure your submission adheres to the PLOS policy on acceptable data access restrictions: <https://journals.plos.org/plosone/s/data-availability#loc-acceptable-data-access-restrictions>.

1) Please confirm whether there are legal or ethical restrictions on sharing your data publicly.

- **Upon checking my University’s Ethics Application and with supervisors, I can confirm that there are ethical restrictions for sharing my qualitative data publicly.**
- **As per my approved Ethics application, I am allowed to use quotes from the interviews but not able to upload the interviews in the official archive at least for 10 years - and only of those participants who consented to upload in public repository. Participants were specifically asked in the consent form if they are ok with making the interviews transcripts available in an official achieve. Most of the participants in the consent form have said "no" to uploading in public repository.**
- **Further, we are not sure about the idea that someone could use the qualitative data to try and replicate findings. Analysis of interviews is a subjective exercise.**
- **Please find the revised data availability statement:**

**"Transcripts (without any personal identifier and of those who consented to sharing of the interview transcripts) are available on request (Email:** [**yoursudesh@gmail.com**](mailto:yoursudesh@gmail.com)**;** [**r.a.page@massey.ac.nz**](mailto:r.a.page@massey.ac.nz)**) after signing confidentiality agreement due to ethical reasons. "**

- **We have further added a condition of signing confidentiality agreement in the data availability statement if anyone request us the data. This is to ensure confidentiality of the stakeholders from Nepal who may be easily identifiable from the transcripts data.**

2) If legal or ethical restrictions apply, please provide all necessary instructions and non-author contact information (preferably email) for a data access committee, ethics committee, or other institutional body that other researchers would require to request access to your data.

**Note that it is not acceptable for an author to be the sole named individual responsible for ensuring data access.**

Please find the email address of all my supervisors who were consulted for the response.

- **As for the data access, we have a process. The primary author is the main contact point as a corresponding author for data access, who will forward the request to all supervisors for consideration. If we do not find any issue with the request collectively, the primary author will send a confidentiality agreement as well as verify the request as genuine. Please note that, we are able to share the data confidentially only of those participants who consented to sharing of the interview data.**
- **Here are the email addresses of my supervisors and Massey University Ethics Committee for your necessary action:**

**Rachel Page: r.a.page@massey.ac.nz
Anna Matheson: anna.matheson@vuw.ac.nz
David Lounsbury: david.lounsbury@einsteinmed.org
James.Faulkner: James.Faulkner@winchester.ac.uk
Danielle Lambrick: D.M.Lambrick@soton.ac.uk
Abhinav Vaidya:** [**dr.abhinavaidya@gmail.com**](mailto:dr.abhinavaidya@gmail.com)

**Massey’s Human Ethics Committee:** [**humanethics@massey.ac.nz**](mailto:humanethics@massey.ac.nz) **(Human Ethics Application SOA 16/37)**

3) If there are no legal or ethical restrictions on sharing your data publicly, please upload the minimal anonymized data set necessary to replicate your study findings as either Supporting Information files, or to a stable public repository. If you upload your data to a repository, please also provide the relevant URLs, DOIs, or accession numbers for other researchers to access your data directly. For a list of recommended public repositories, please see <https://journals.plos.org/plosone/s/recommended-repositories>.

- **As mentioned above, ethical restrictions apply**
- **Here is the updated Data availability statement:**

**"Transcripts (without any personal identifier and of those who consented to sharing of the interview transcripts) are available on request (Email:** [**yoursudesh@gmail.com**](mailto:yoursudesh@gmail.com)**;** [**r.a.page@massey.ac.nz**](mailto:r.a.page@massey.ac.nz)**) after signing a confidentiality agreement due to ethical reasons. "**
