# Supplementary material for: Dietary practices, physical activity and social determinants of non-communicable diseases in Nepal: A systemic analysis
Source: PLoS One. 2023 Feb 6;18(2):e0281355. doi: 10.1371/journal.pone.0281355 (PMC9901760; doi:10.1371/journal.pone.0281355)
Supplement: Attachment — Submitted filename: Response to reviewers.docx [file pone.0281355.s005.docx]

# **Response to reviewers**

***Journal Requirements:

Please review your reference list to ensure that it is complete and correct. If you have cited papers that have been retracted, please include the rationale for doing so in the manuscript text, or remove these references and replace them with relevant current references. Any changes to the reference list should be mentioned in the rebuttal letter that accompanies your revised manuscript. If you need to cite a retracted article, indicate the article’s retracted status in the References list and also include a citation and full reference for the retraction notice.***

- Thank you for your suggestion. We authors have reviewed and checked the references and links.

***Additional Editor Comments:

Abstract
Incomplete sentence, add diet and physical activity after determinants
....interactions of the social determinants ....***

- We have added “of NCDs” as we are being consistent throughout the article.

***Can the authors provide how this can be achieved:
The health system has potential to play a more effective role in the prevention of the behavioural and social determinants of NCDs.***

- This statement is from abstract. We authors have elaborated this in the conclusion section. As indicated within the paper, curative orientation and health system management issues as well as political and commercial influences outside of the health system are creating systemic barriers for effective multi-sectoral action for the prevention of NCDs risk factors. The first and most important shift needed is creating and resourcing an appropriate prevention structure (a national centre) for acclerating actions on the SDH.

***I have made a few comments (as HTML markup)***

- Thank you for your suggestion. We authors have reviewed and revised the manuscript as per HTML markup as follows:
- Line 7 strikeout: changed percentage and statement so that the contrasting argument is more clearer i.e. “but 97% had adequate physical activity”
- Line 10: deleted as suggested i.e. “indicating the shifts towards physical inactivity in Nepal”
- Line 53-59: As suggested, further clarified rationale for selecting the districts i.e. “The study was conducted in Nepal from July until October 2016. While there was limited district specific data on the prevalance of NCDs, the nation surveys showed an increasing trend in both urban and rural areas throughout Nepal [4, 28]. Two districts (Bhaktapur and Morang) were purposively selected as case districts, based on first author’s prior relationships with respective district health offices and place. In particular, the first author is from Morang and his motivation to study about social determinants of NCDs grew from his personal experience of observing family and wider community being impacted by NCDs.”
- Line 170: Nepali term “Dhido” explained within large bracket i.e. “thick porridge made from corn or buckwheat flour”
